# Atorvastatin suppresses HIV/antiretroviral drug–induced cardiac fibrosis and dysfunction in mice by blocking platelet TGF-**β**1 signaling

**DOI:** 10.1172/jci.insight.198647

**Published:** 2026-02-05

**Authors:** Kumar Subramani, Denys Babii, Brienne Cole, Tayyab A. Afzal, Thamizhiniyan Venkatesan, Trevor Word, Sandra Gostynska, Sixia Chen, Kar-Ming Fung, Ali Danesh, Itzayana G. Miller, Paul Klotman, Brad R. Jones, Jeffrey Laurence, Jasimuddin Ahamed

**Affiliations:** 1 Cardiovascular Biology Research Program, Oklahoma Medical Research Foundation, Oklahoma City, Oklahoma, USA.; 2 University of Oklahoma Health Sciences Center, Oklahoma City, Oklahoma, USA.; 3 Division of Infectious Diseases, Weill Cornell Medical College, New York, New York, USA.; 4 Baylor College of Medicine, Houston, Texas, USA.; 5 Division of Hematology and Medical Oncology, Weill Cornell Medical College, New York, New York, USA.

**Keywords:** AIDS/HIV, Cardiology, Fibrosis

## Abstract

Cardiovascular disease (CVD) contributes to morbidity and mortality in people with HIV (PWH) receiving antiretroviral therapy (ART). In the REPRIEVE trial, pitavastatin reduced atherosclerotic CVD risk to a magnitude inconsistent with pitavastatin’s impact solely on LDL cholesterol and inflammation. Here, atorvastatin and ART used in REPRIEVE, including tenofovir, emtricitabine, and dolutegravir, ritonavir and darunavir were examined in 2 mouse models: transgenic HIV-Tg26 mice and HIV-PDX mice engrafted with T cells from PWH. HIV-Tg26 and HIV-PDX mice had higher cardiac fibrosis than littermate controls without HIV. Administration of tenofovir, emtricitabine, and dolutegravir or ritonavir, but not darunavir, resulted in an approximately 2-fold increase in fibrosis. Mice depleted of platelet TGF-β1 or treated with atorvastatin were partially protected from HIV- and ART-induced cardiac fibrosis, steatosis, and diastolic dysfunction. Atorvastatin’s effects were independent of changes in inflammatory cytokines, which correlated with reduced platelet activation and TGF-β signaling in cardiac endothelial cells, fibroblasts, and macrophages undergoing mesenchymal transition. Our results indicate that certain ART regimens accelerate HIV-associated CVD characterized by heart failure with preserved ejection fraction via platelet TGF-β1–dependent processes, which were mitigated by atorvastatin. Our findings provide a potential mechanism for the pleiotropic effects of statins in HIV/ART-linked CVD, which could be targeted by antiplatelet agents or inhibition of TGF-β signaling.

## Abstract 2

· Contemporary ART regimens induce release of platelet TGFβ1 and are associated with cardiac fibrosis and diastolic dysfunction with ectopic fat deposition in HIV-infected mice. · Depleting platelet TGFβ1 and/or treating with atorvastatin therapy suppresses HIV-ART-induced cardiac fibrosis, suggesting use of anti-platelet strategies to prevent heart failure among PWH.

## Introduction

HIV infection has been linked to several non–AIDS-defining illnesses, particularly cardiovascular disease (CVD). This contributes significantly to morbidity and mortality in people with HIV (PWH) despite effective antiretroviral therapy (ART) (1). A major advance in terms of prevention derives from the Randomized Trial to Prevent Vascular Events in HIV (REPRIEVE), demonstrating that pitavastatin decreased atherosclerotic CVD among ART-treated PWH (2). The observed reduction was much greater than predicted based on the magnitude of decrease in LDL cholesterol achieved (2). In terms of potential mechanisms, pitavastatin had no effect on other non–AIDS-defining comorbidities which, like atherosclerotic CVD, have been linked to chronic inflammation among ART-treated PWH, including end-stage liver disease, end-stage renal disease, tuberculosis, and malignancy (2, 3). Indeed, although a decrease in lipoprotein-associated phospholipase A2, a marker of arterial inflammation, was reported among REPRIEVE participants receiving pitavastatin, there was no change in other proinflammatory biomarkers, including high-sensitivity C-reactive protein (hsCRP), MCP-1, sCD14, CD163, IL-1β, IL-6, IL-10, IL-18, and caspase-1 (3).

A recent secondary analysis of REPRIEVE found that pitavastatin increased procollagen C-endopeptidase enhancer 1 (PCOLCE), which enhances activity of proteinases essential for the formation and assembly of vascular extracellular matrix (4). Those processes may be associated with the transformation of vulnerable plaque phenotypes to more stable coronary lesions (4), consistent with the impact of pitavastatin on early atherosclerotic cardiovascular events in that trial. This is similar to the protective effects of the profibrotic cytokine TGF-β1 in early atherosclerosis, which promotes collagen synthesis and plaque stability, although the opposite effects have been suggested in later stages of atherosclerosis, with plasma TGF-β1 levels significantly increased in patients with coronary artery ectasia and coronary artery disease (5). However, emerging data related to HIV-linked CVD in general, and the heart failure mechanisms and phenotypes that predominate among PWH in the contemporary ART era, have the potential to further modify CVD treatment and prevention strategies (6). With this concern, we sought preclinical evidence on the ability of statins to influence heart failure with preserved ejection fraction (HFpEF), which is characterized by myocardial fibrosis and steatosis in both PWH and the general population (7–11). It is now recognized in 35% of CVD cases in PWH on ART (6).

Disentangling the effects of HIV versus ART on myocardial structural and functional pathology through patient-oriented research is challenging, given the potential divergent effects of different ART drugs and drug classes (12–15). In our study, we leveraged 2 established mouse models of HIV: (a) transgenic Tg26 mice expressing 7 HIV proteins but producing no infectious virus (HIV-Tg26) (16) and (b) HIV patient-derived xenograft (HIV-PDX) mice, which are nonobese diabetic SCID IL2rγ^null^ mice engrafted with HIV-infected memory CD4^+^ T cells from PWH (17). We exposed these mice to ART drugs used in REPRIEVE, including a nucleoside/nucleotide and integrase strand transfer inhibitor–based (INSTI-based) regimen as well as protease inhibitors used in protease inhibitor–boosted regimens. Both INSTI- and protease inhibitor–based ART are associated with an increased risk of many forms of CVD compared with non-nucleoside reverse transcriptase inhibitor–based ART (13–15, 18–21). We characterized myocardial structural and functional pathology at 8 weeks after treatment. We also tested whether atorvastatin, a lipophilic statin in the same class as pitavastatin, could protect cardiac structure/function in the same settings and explored the mechanisms underlying such preservation. These data suggest a potential pathway by which statins influence specific forms of CVD in ART-treated PWH. This mechanism appears to operate independently of changes in total cholesterol and inflammatory cytokines, aligning with observations from the REPRIEVE trial.

## Results

### HIV-Tg26 and HIV-PDX mice have higher cardiac fibrosis than littermate controls without HIV — an INSTI-based ART regimen and the protease inhibitor RTV further increased cardiac fibrosis.

**Figure 1:**
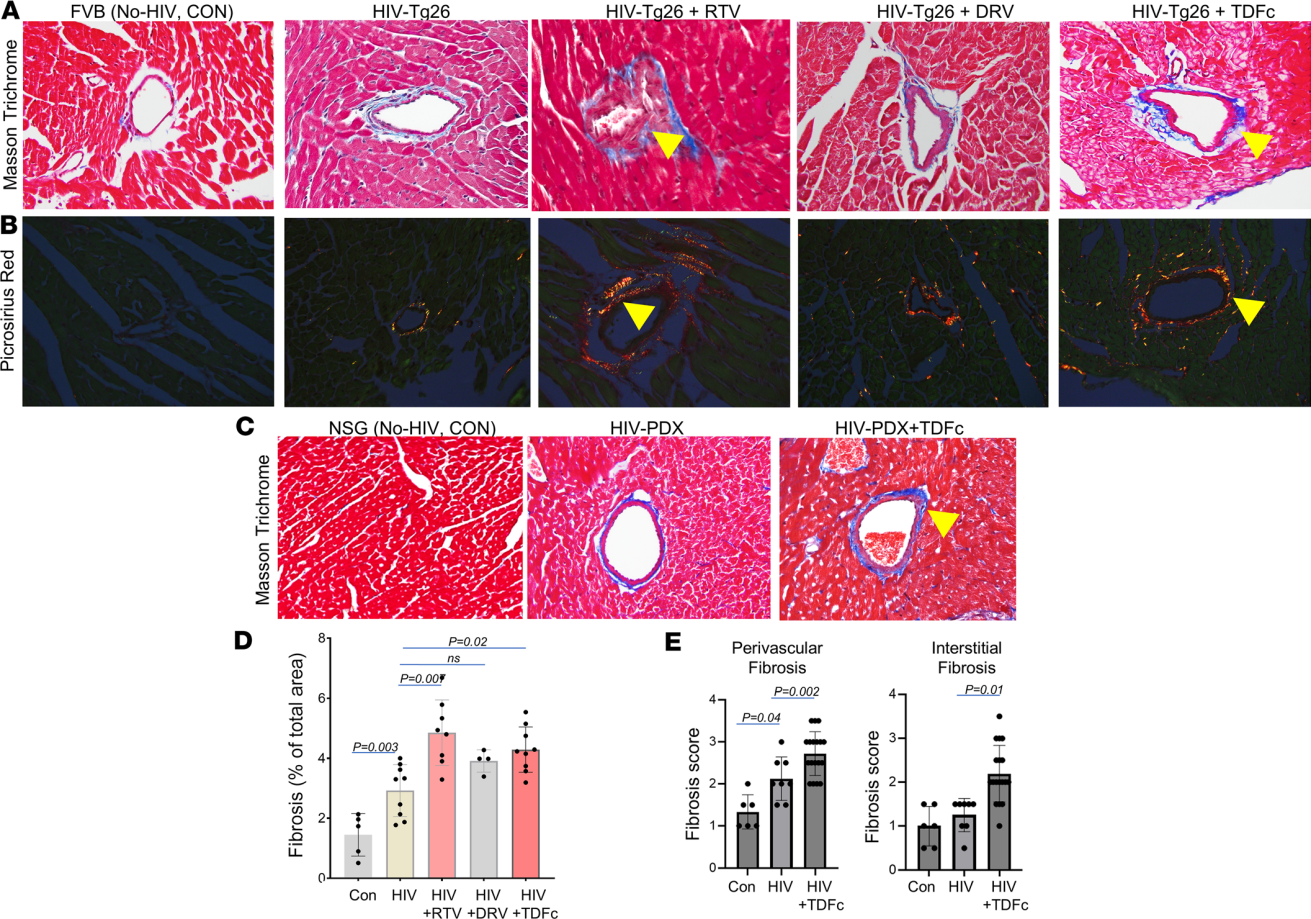
Effect of ART on cardiac fibrosis in HIV mice. (**A**) Representative images of Masson’s trichome staining of heart sections from FVB/NJ -No HIV controls, HIV-Tg26 mice, and HIV-Tg26 mice treated with ART ritonavir (RTV), darunavir (DRV), or the tenofovir-emtricitabine-dolutegravir (TDF-FTC-DTG) cocktail (TDFc) for 8 weeks. Excessive collagen is indicated in blue. (**B**) Representative images of Picrosirius red staining, showing fibrotic areas as a mixture of green, red, and yellow, under polarized light microscopy. (**C**) Representative images of Masson’s trichome staining of heart sections from NSG controls, HIV-infected (HIV-PDX) mice, and HIV-PDX mice treated with TDFc for 8 weeks. (**D**) Quantification of fibrotic areas in Masson’s trichome–stained and Picrosirius red images showed that HIV-infected mice had higher cardiac fibrosis than controls. ART-treated HIV-Tg26 mice, with the exception of those treated with DRV, had more fibrotic areas than untreated ones. (**E**) Blind scoring for fibrosis by microscopy on a 0–4 scale showed that ART-treated mice exhibited higher perivascular and interstitial fibrosis than untreated HIV-PDX control mice. Both patterns of fibrosis were quantified from whole-heart Masson’s trichome–stained and Picrosirius red images acquired from a slide scanner (Supplemental Figures 1, A–C). In **D** and **E**, total, perivascular, and interstitial fibrosis were quantified using the algorithm shown in Supplemental Figure 1A or blindly by a pathologist and a trained technician who were unaware of mouse genotype. Each dot in the bar graphs represents an individual mouse; data are presented as mean ± SD; *P* values < 0.05 were considered significant using standard 2-tailed Student’s *t* test.

We previously showed that WT C57BL/6 mice treated with supratherapeutic doses of ritonavir (RTV) had an approximately 3-fold increase in cardiac fibrosis compared with untreated mice (~1.5% vs. ~0.5%) (22). PWH treated with protease inhibitor–based ART appear at greatest risk for CVD (14, 15), including cardiac fibrosis. Thus, we first tested whether HIV alone is associated with enhanced cardiac fibrosis in mice and then whether doses of RTV equivalent to that used clinically in RTV-boosted protease inhibitor regimens, or a common INSTI-based ART regimen, tenofovir (TDF), emtricitabine (FTC), and dolutegravir (DTG), enhance such fibrosis. Only 2 of 10 (20%) WT littermate control mice on an FVB/NJ, C57BL/6, or NSG background had mild cardiac fibrosis (~1.5% of the total cardiac area) at 4 months of age, as measured by Masson’s trichome and Picrosirius red staining. In contrast, 8 of 11 (73%) of HIV-Tg26 mice — those containing 7 HIV-1 genes but incapable of producing infectious virus — developed mild to moderate fibrosis (2%–4% of the total cardiac area). Administration of RTV or TDF-FTC-DTG, i.p. daily for 8 weeks, led to moderate cardiac fibrosis (3%–4% of the total cardiac area) in 12 of 15 (80%) of these mice. (*P* = 0.003 for WT control vs. HIV-Tg26 mice and *P* = 0.007 for HIV-Tg26 vs. RTV-treated HIV-Tg26 mice) (Figure 1, A, B, and D, and Supplemental Figure 2; supplemental material available online with this article; https://doi.org/10.1172/jci.insight.198647DS1).

As a control, we challenged HIV-Tg26 (Figure 1, A, B, and D) and WT C57BL/6 (Supplemental Figure 2) mice with clinically relevant doses of darunavir (DRV) alone. This protease inhibitor has not been linked clinically to CVD in the absence of RTV boosting (14). We found no elevation of cardiac fibrosis over pretreatment levels in either HIV-Tg26 or WT mice. Hematological values (including RBC, WBC, and platelet count) showed that Tg26 mice were mildly anemic and thrombocytopenic, but all other parameters were similar to those observed in control mice (Table 1). Platelet aggregation responses induced by ADP or thrombin were also similar in platelets isolated from Tg26 and control mice (data not shown).

Comparable results were observed in HIV-PDX mice infected with HIV with and without TDF-FTC-DTG treatment (Figure 1, C–E). Nine of 11 (82%) mice exhibited both perivascular and interstitial fibrosis with TDF-FTC-DTG treatment compared with 40% of HIV-PDX mice not exposed to these drugs, as assessed by Masson’s trichome and Picrosirius red staining and imaging under normal and polarized light, respectively (Figure 1, C–E).

### Both an INSTI-based ART regimen and RTV induce diastolic dysfunction with preserved EF in HIV mice.

**Figure 2:**
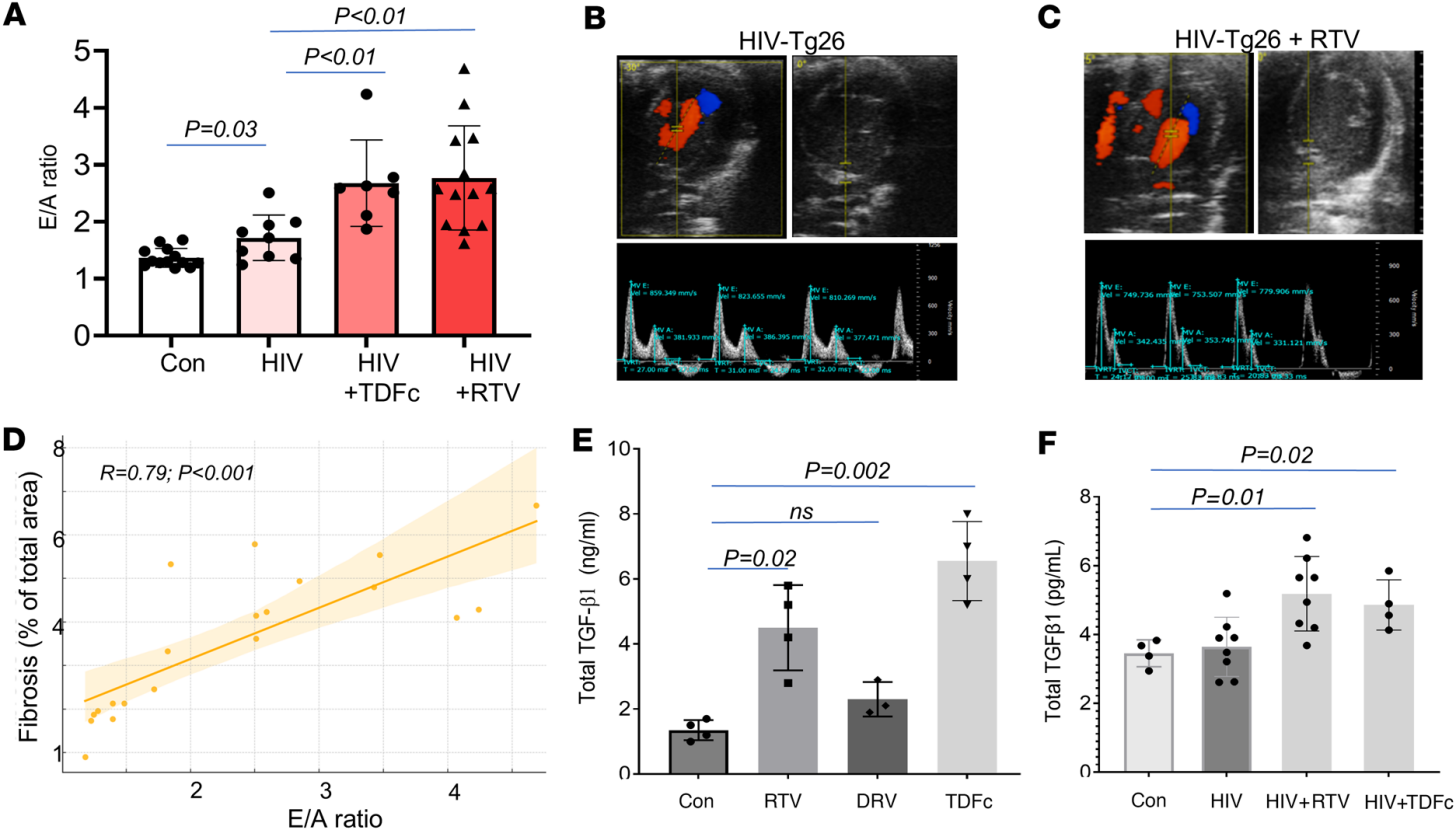
Effect of ART on cardiac function in HIV-Tg26 mice. (**A**) Bar plot showing increased early-to-late (E/A) ventricular filling velocity ratios indicating impaired diastolic function after 8 weeks of exposure to RTV or the TDF-FTC-DTG cocktail (TDFc) compared with untreated HIV-Tg26 control mice. (**B** and **C**) Representative ultrasound images (Vevo2100) showing 4-chamber views with color-Doppler (upper left; blood flow in red) across the mitral valve annulus (upper right) and power-Doppler (lower panels; peak E and A wave velocities) in HIV mice without (**B**) or with (**C**) RTV treatment for 8 weeks. (**D**) E/A ratios directly correlated with cardiac fibrosis in control and ART-treated HIV mice. (**E** and **F**) Bar plots showing total TGF-β1 levels as measured by ELISA in (**E**) platelet releasates (10^9^ per mL) prepared from healthy human volunteers before and after stimulation with RTV (5 μM), darunavir (DRV; 15 μM), or TDFc. (**F**) Plasma prepared from HIV-Tg26 mice treated with RTV or TDFc. Each dot in the bar graphs represents an individual mouse; data are presented as mean ± SD; *P* values < 0.05 were considered significant using standard Student’s *t* test.

To assess whether the degree of fibrosis seen in ART-treated HIV mice was of functional significance, we evaluated cardiac function by echocardiography. Systolic dysfunction was not observed based upon preservation of both EF and fractional shortening in WT and TDF-FTC-DTG–treated HIV-Tg26 mice (Supplemental Figure 3). Diastolic function in HIV-Tg26 mice was slightly impaired compared with HIV-negative WT controls in the absence of ART (*P* = 0.03). More severe diastolic dysfunction was noted in both INSTI- and RTV-challenged HIV-Tg26 mice compared with non–drug-treated HIV-Tg26 mice, which manifested as higher early-to-late (E/A) ventricular filling velocity ratios (Figure 2, A–C; *P* < 0.01). There was a significant correlation between cardiac fibrosis and diastolic indices, specifically E/A ratios, in HIV-Tg26 mice with and without ART compared with control Tg26 mice (Figure 2D).

In terms of potential mechanisms, we previously showed that RTV induces release of TGF-β1 in vitro from human platelets (21). In the present study, we showed that RTV alone, as well as the TDF-FTC-DTG cocktail, induced release of TGF-β1 from freshly isolated human platelets, whereas the protease inhibitor DRV had no effect (Figure 2E). RTV and TDF-FTC-DTG also induced release of TGF-β1 in vivo as assessed in plasma from ART-treated HIV-Tg26 mice (Figure 2F).

### ART-induced ectopic fat deposition in the heart is associated with cardiac fibrosis in HIV mice.

**Figure 3:**
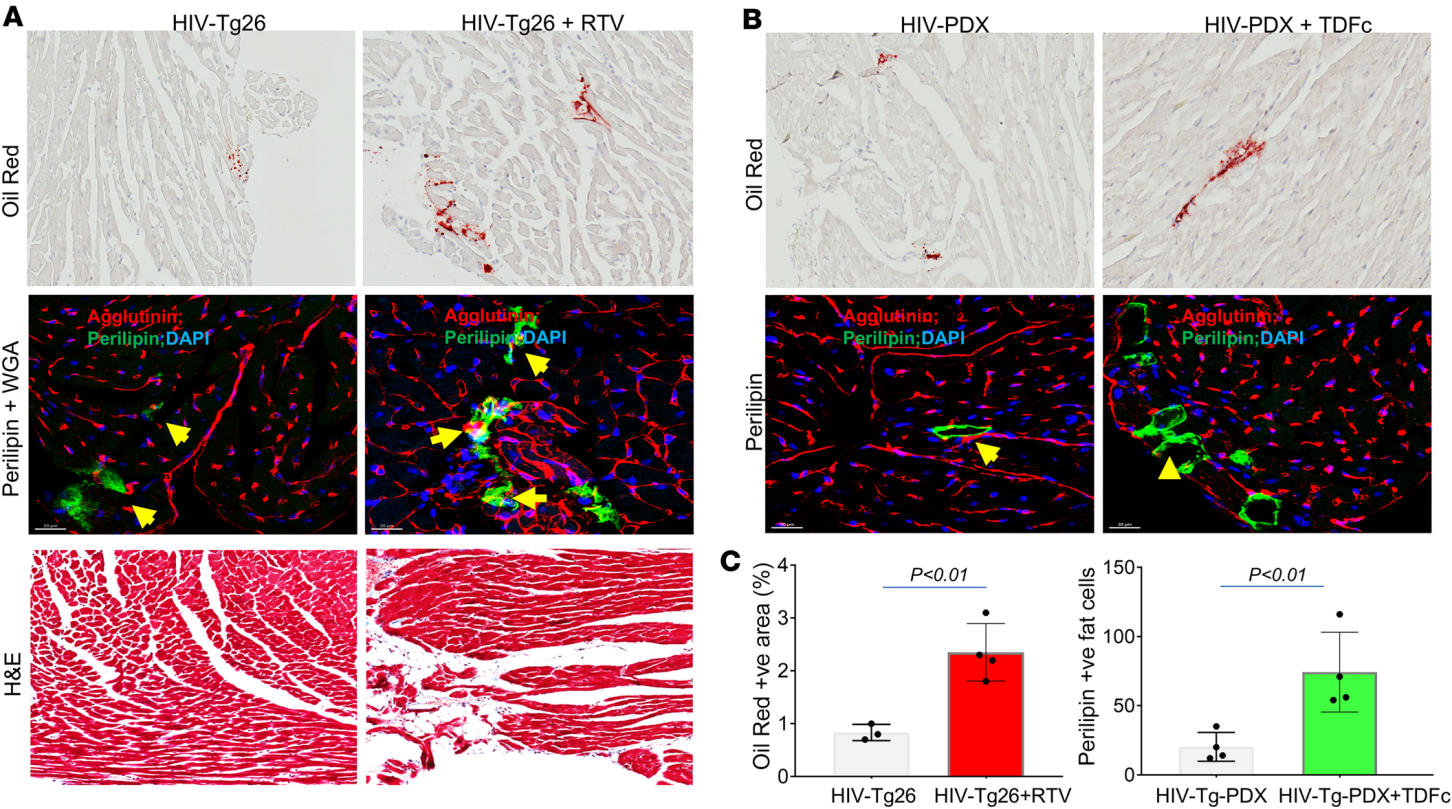
ART-induced ectopic fat deposition in the heart is associated with cardiac fibrosis in HIV-Tg26 and HIV-PDX mice. (**A** and **B**) HIV-Tg26 and HIV-PDX mice were challenged with RTV or TDF-FTC-DTG. Oil Red O–positive areas indicate lipid/fat cell deposition in the heart (upper panels), which were confirmed by perilipin immunofluorescence with confocal microscopy (middle panels) and by H&E staining (**A**, lower panels). Oil Red O–positive areas were also matched with fibrotic areas. Scale bars: 20 µm (**C**) Quantification of lipid (left) or fat cells (right) from Oil Red O–positive or perilipin-positive regions, respectively, in the hearts of ART-treated HIV-Tg26 and HIV-PDX mice. Each dot in the bar graphs represents an individual mouse; data are presented as mean ± SD; *P* values < 0.05 were considered significant using standard 2-tailed Student’s *t* test.

Cardiac fat deposition is increased in ART-treated PWH and is associated with diastolic dysfunction (18, 19). However, its relationship to cardiac fibrosis in the setting of HIV/ART has not been examined. We evaluated the effect of RTV and TDF-FTC-DTG on fat accumulation in hearts of HIV-Tg26 and HIV-PDX mice. Both ART regimens resulted in higher cardiac fat deposition, as shown by Oil Red O staining (Figure 3, A and B). To determine whether these stained areas contained fat cells, they were immunostained with an antibody to perilipin, a marker of fat cells, which revealed clusters of perilipin-positive fat cells (Figure 3, A and B).

The same heart sections were then stained with Picrosirius red, Masson’s trichome, and H&E. Fibrotic areas with excessive collagen accumulation surrounded by fat cells were recognized in superimposed images (Figure 3A). Quantification confirmed the association between fibrosis and lipid/fat cell accumulation in ART-treated hearts of both HIV-Tg26 and HIV-PDX mice (Figure 3C). Taken together, these results indicate that ART-treated HIV mice with higher cardiac fibrosis also have greater cardiac fat cell deposition.

### Platelet-derived TGF-β1 contributes to RTV-induced cardiac fibrosis and fat cell deposition.

**Figure 4:**
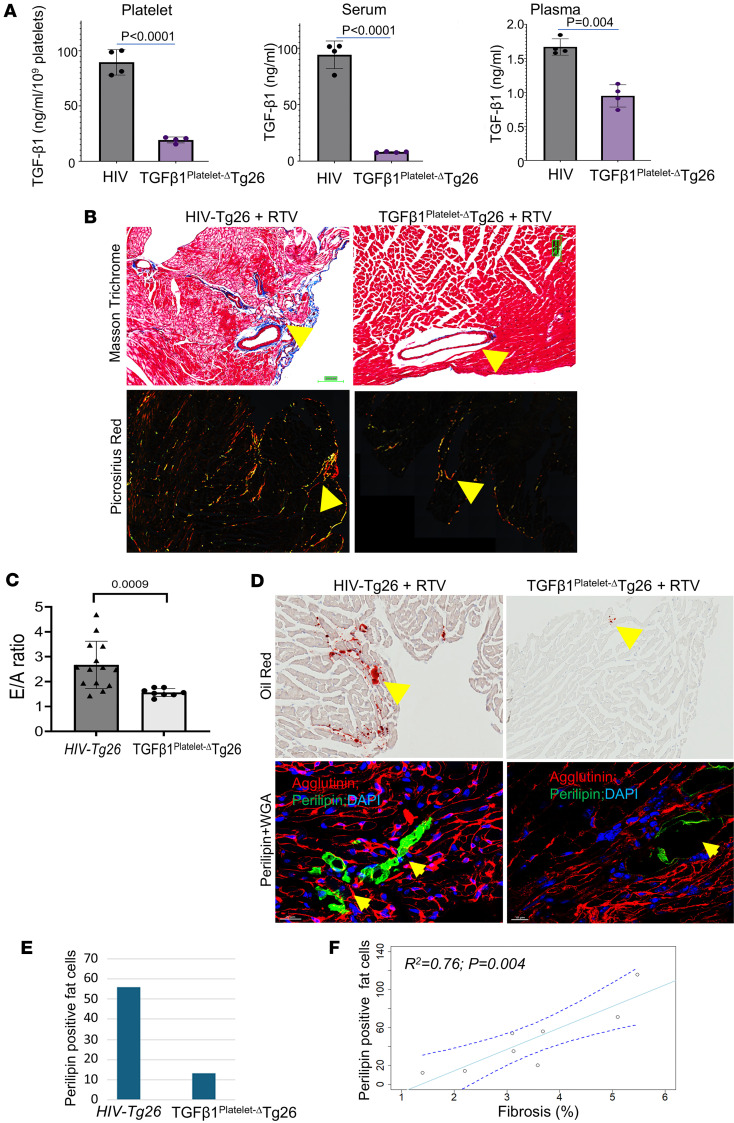
TGF-β^1Platelet-^ΔTg26 mice are partially protected from developing RTV-induced cardiac fibrosis and deterioration of diastolic function. (**A**) TGF-β1^Platelet-^ΔTg26 mice (purple) showed a more than 90% decrease in TGF-β1 in platelets and serum, and a more than 50% decrease in plasma TGF-β1, compared with littermate control HIV-Tg26 mice (gray), as measured by ELISA (*n* = 4). (**B**) Images of heart sections from RTV-challenged HIV-Tg26 or TGF-β1^Platelet-^ΔTg26 mice were captured under polarized light, revealing fibrotic areas in blue in Masson’s trichrome staining and as mixtures of green, red, and yellow in Picrosirius red staining. TGF-β1^Platelet-^ΔTg26 mice accumulated lower collagen levels than HIV-Tg26 mice, as confirmed by quantification of Picrosirius red staining, which revealed smaller fibrotic areas in TGF-β1^Platelet-^ΔTg26 mice (2.5% ± 0.5%) compared with HIV-Tg26 mice (3.3% ± 0.7%; *P* < 0.01 by Student’s *t* test). Scale bars: 200 μm. (**C**) E/A ratios measured by echocardiography, showing protection from impairment of diastolic dysfunction in TGF-β1^Platelet-^ΔTg26 mice (light gray). (**D**) Oil Red O and perilipin staining show reduced fat cell accumulation in heart tissue from RTV-challenged TGF-β1^Platelet-^ΔTg26 mice compared with RTV-challenged HIV-Tg26 mice. (**E**) Bar plot showing the number of perilipin-positive fat cells per heart in RTV-challenged TGF-β1^Platelet-^ΔTg26 and HIV-Tg26 mice, quantified from confocal microscopy images. Each bar represents the average of 2 heart sections. (**F**) Correlation plot showing the positive association between cardiac fibrosis and fat cell deposition in the hearts of HIV-Tg26 mice and TGF-β1^Platelet-^ΔTg26 mice challenged with RTV.

We previously showed that platelet TGF-β1 contributes to RTV-induced cardiac fibrosis in WT C57BL/6 mice (22). To test whether platelet TGF-β1 contributes to cardiac fibrosis in mice treated with various ART drugs in the context of HIV, we generated HIV-Tg26 mice with platelet TGF-β1–deficient (TGF-β1^Platelet-Δ^Tg26) by crossing HIV-Tg26 mice with PF4CreTgfb1^fl/fl^ mice. We found a more than 90% decrease in total TGF-β1 in platelets and serum and a 50% decrease in plasma of TGF-β1^Platelet-Δ^Tg26 mice compared with littermate control HIV-Tg26 mice (Figure 4A). This indicates that platelets were the major source of circulating TGF-β1 in HIV mice. Although platelets are the most abundant source of TGF-β1, other cells, including macrophages, DCs, Tregs, and B cells, could also produce TGF-β1 (23).

We observed reduced levels of cardiac fibrosis in RTV-treated TGF-β1^Platelet-Δ^Tg26 mice (2.1% ± 0.25%) compared with RTV-treated HIV-Tg26 littermate mice (4.68% ± 0.85%; *P* < 0.01) (Figure 4B). The percentage of areas of fibrosis was 2.1% ± 0.25% in TGF-β1^Platelet-Δ^Tg26 mice versus 4.68% ± 0.85% in HIV-Tg26 mice (*P* < 0.01). Reduced fibrosis in RTV-treated TGF-β1^Platelet-Δ^Tg26 mice was accompanied by protection from diastolic dysfunction (Figure 4C). Since ART-induced accumulation of lipid/fat cells in the heart correlated with fibrosis, we also assessed lipid/fat cell accumulation in TGF-β1^Platelet-Δ^Tg26 mice. Staining of RTV-treated TGF-β1^Platelet-Δ^Tg26 mouse hearts with Oil Red O revealed lower lipid accumulation compared with HIV-Tg26 littermates challenged with RTV. Immunostaining with perilipin confirmed less accumulation of fat cells in these mice compared with littermate controls (Figure 4, D and E). The presence of fat cells correlated with levels of fibrosis in TGF-β1^Platelet-Δ^Tg26 and HIV-Tg26 littermate control mice challenged with RTV (Figure 4F).

To test whether ART-associated release of TGF-β1 in vivo is likely to be primarily derived from platelets, we challenged TGF-β1^Platelet-Δ^Tg26 mice with RTV and TDF-FTC-DTG. No increase in TGF-β1 levels over baseline was observed (data not shown), indicating that ART increases TGF-β1 in vivo primarily by activating platelets.

### Atorvastatin suppresses TGF-β signaling, cardiac fibrosis and fat accumulation, and diastolic dysfunction without altering inflammatory cytokines and total cholesterol in HIV mice.

**Figure 5:**
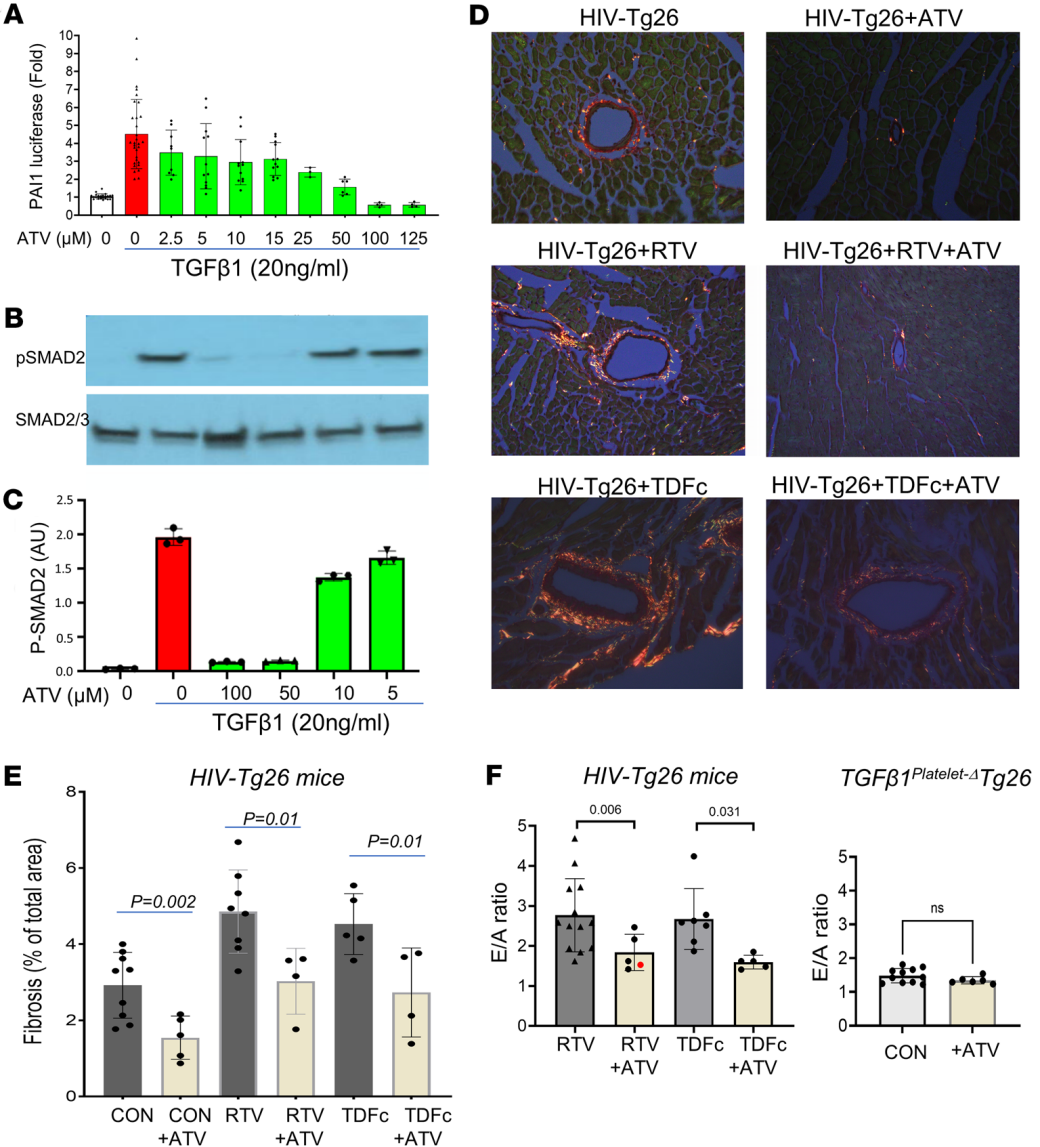
Atorvastatin suppresses TGF-β1 signaling, cardiac fibrosis, and fat cell deposition and improves diastolic dysfunction in HIV mice. (**A**) Atorvastatin (ATV) inhibited TGF-β1–mediated PAI1 luciferase activity and (**B**) SMAD2 phosphorylation in a dose-dependent manner in mink lung epithelial cells stimulated with platelet TGF-β1 (20 ng/mL) for 16–18 hours. (**C**) Quantification of dose-dependent inhibition of SMAD2 phosphorylation levels by ATV. (**D**) Picrosirius red staining of heart sections from HIV-Tg26 mice challenged with vehicle, ATV, RTV, or the TDF-FTC-DTG cocktail (TDFc) for 8 weeks, showing that ATV halted cardiac fibrosis, as visualized under polarized light. Original magnification ×20. (**E**) Quantification of Picrosirius red staining showing significantly lower fibrosis in ATV-cotreated mice compared with mice exposed to vehicle, RTV, or TDFc alone. Group comparisons were performed using Wilcoxon’s rank-sum test. (**F**) E/A ratios measured by echocardiography, showing lower impairment of diastolic function in HIV-Tg26 mice cotreated with ATV (yellow) compared with those exposed to RTV or TDFc alone. (**G**) Bar plot showing no significant difference in diastolic functions (as measured by E/A ratio) in combined PF4-Cre (*n* = 3) and G1b-Cre (*n* =3) Tgfb^1fl/fl^ (TGF-β^1Platelet-^ΔTg26) mice treated with ATV for 8 weeks (light gray). Each dot represents Masson’s trichome staining or Picrosirius red quantifications from whole heart images from an individual mouse (Supplemental Figure 1, A–C); data are presented as mean ± SD; *P* values < 0.05 were considered significant using standard Student’s *t* test.

The REPRIEVE trial showed that pitavastatin, a lipophilic statin, had a beneficial effect in reducing atherosclerotic CVD (2). We first tested whether another lipophilic statin, atorvastatin, could influence processes related to the second major type of CVD linked clinically to HIV/ART, fibrosis-associated HFpEF, by inhibiting TGF-β1–induced signaling. We used an engineered cell culture system in which TGF-β1 stimulation induces PAI1 luciferase activity in mink lung epithelial cells (MLECs) expressing a TGF-β–responsive PAI1 reporter fused with a luciferase gene (24). This signaling response was inhibited by atorvastatin in a dose-dependent manner (Figure 5A). This is consistent with the finding that TGF-β1–induced SMAD2/3 phosphorylation signaling was inhibited by atorvastatin, also in a dose-dependent manner (Figure 5, B and C). Atorvastatin also inhibited TGF-β1–induced signaling responses assessed by PAI1 and SMAD phosphorylation in vitro in the presence of RTV or TDF-FTC-DTG (Supplemental Figure 5, A–C).

To investigate whether atorvastatin could prevent HIV-linked cardiac fibrosis in HIV mice in vivo, we administered 3 mg/kg in drinking water to HIV-Tg26 mice and measured cardiac fibrosis and heart function. This dose corresponds to 20 mg/d in an 80 kg human and is well tolerated in mice, without toxicity (25, 26). Less cardiac fibrosis and impairment of diastolic function was observed compared with untreated HIV-Tg26 mice over an 8-week period (Figure 5, D and E). Atorvastatin administration along with TDF-FTC-DTG or RTV to HIV-Tg26 mice also reduced cardiac fibrosis and preserved diastolic function compared with ART-exposed HIV-Tg26 mice not receiving atorvastatin (Figure 5, D–F, and Supplemental Figure 6). Treatment of TGF-β1^Platelet-Δ^Tg26 mice with atorvastatin did not further deteriorate diastolic function compared with basal levels (Figure 5G), with no change in cardiac fibrosis levels (2.0% ± 0.2% in TGF-β1^Platelet-Δ^Tg26 mice vs. 2.1% ± 0.5% in atorvastatin-treated mice), indicating that atorvastatin inhibits cardiac fibrosis and diastolic dysfunction by blocking platelet-TGF-β1–induced signaling.

Atorvastatin did not alter inflammatory cytokines IL-6 and TNF-α, PPAR-γ, or plasma total cholesterol and glucose levels in HIV-Tg26 mice with or without ART (Supplemental Figure 4 and Supplemental Figure 9). Staining of heart sections with Masson’s trichome, Picrosirius red, or Oil Red O and perilipin revealed lower fibrosis and lipid/fat cell accumulation in atorvastatin-treated versus vehicle treated mice challenged with TDF-FTC-DTG or RTV (Supplemental Figure 6). Collectively, these results indicated that atorvastatin inhibits cardiac fibrosis and diastolic dysfunction by blocking platelet TGF-β1–induced signaling.

### Atorvastatin inhibits platelet activation and TGF-β signaling and suppresses myofibroblast transition and collagen expression in cardiac cells.

**Figure 6:**
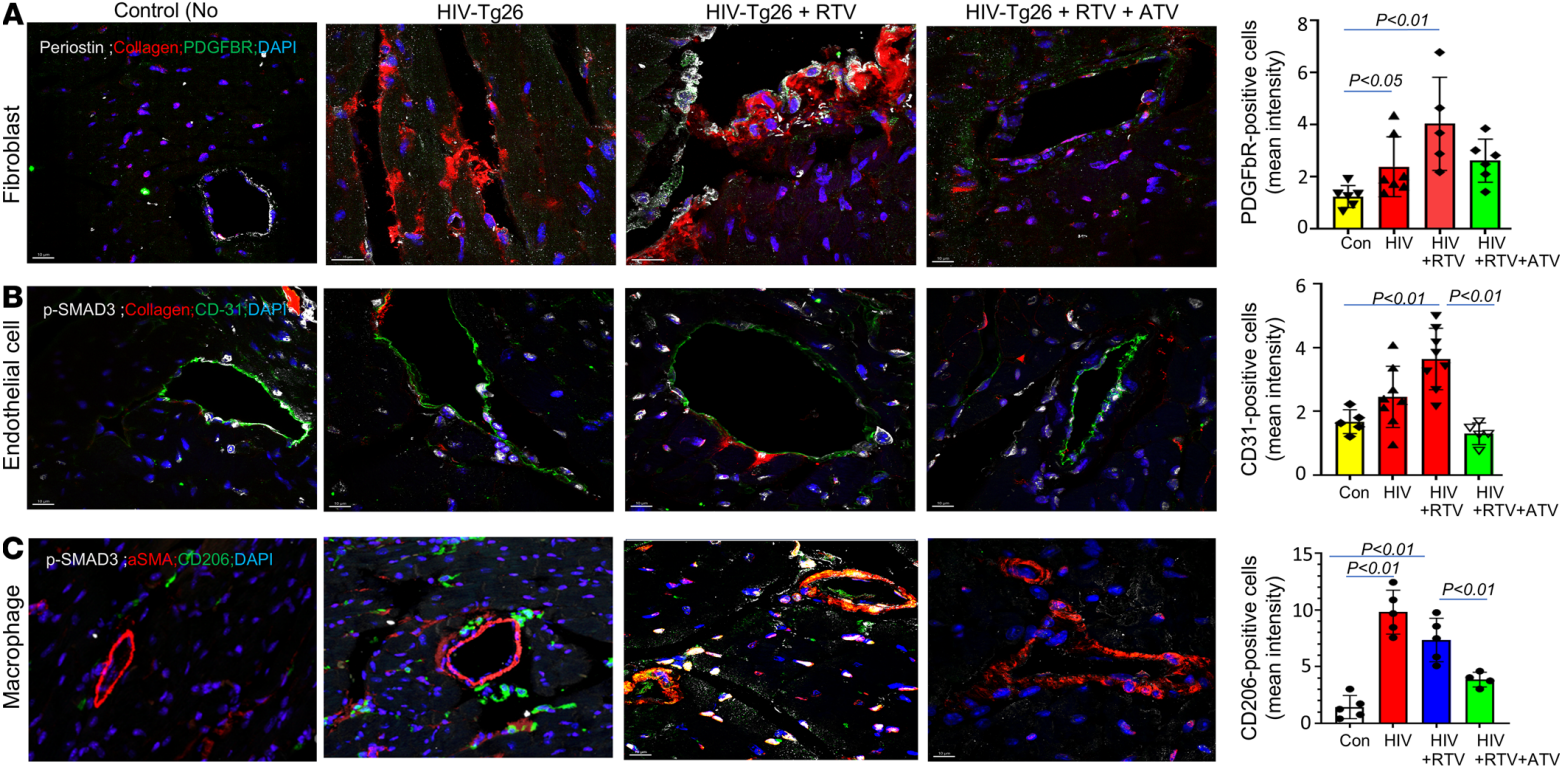
Atorvastatin inhibits SMAD signaling and mesenchymal transition in the hearts of HIV-Tg26 mice. Confocal microscopy images and immunofluorescence quantification of myofibroblast markers PDGFβR (fibroblasts) (**A**), CD31 (endothelial cells) (**B**), and CD206 (macrophages) (**C**) in the hearts of HIV-negative control mice, untreated, and RTV-treated HIV-Tg26 mice, and atorvastatin plus RTV cotreated HIV-Tg26 mice. Each dot in the bar graphs represents an individual mouse; data are presented as mean ± SD; *P* values < 0.05 were considered significant using standard 2-tailed Student’s *t* test. Scale bars: 10 μm

**Figure 7:**
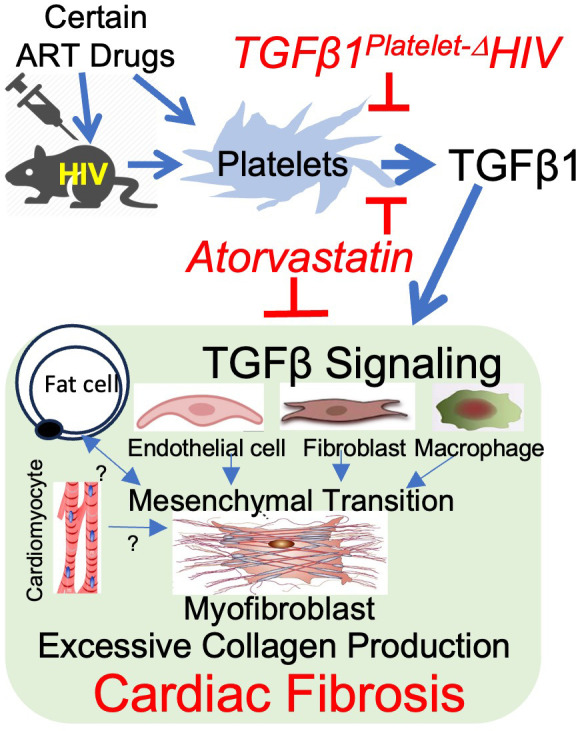
Graphic model showing that HIV-ART induces the release of TGF-β1 from platelets, which activates downstream TGF-β signaling in various cardiac cell types. This drives the mesenchymal transition of these cardiac cells into myofibroblasts, which produce excessive collagen, leading to cardiac fibrosis. These responses can be mitigated by atorvastatin administration, which inhibits TGF-β signaling and platelet activation.

Atorvastatin inhibits platelet activation induced by classic platelet agonists (27). We showed that atorvastatin inhibited human platelet activation in vitro and reduced plasma TGF-β1 levels in mice cotreated with certain ART drugs (Supplemental Figure 7). To test whether atorvastatin blocks TGF-β1 signaling in vivo, we performed multicolor immunofluorescence and confocal microscopy on HIV-Tg26 mouse hearts with and without atorvastatin treatment and in mice deficient in platelet TGF-β1 (TGF-β1^Platelet-Δ^Tg26). HIV-Tg26 mice had higher TGF-β1 signaling, as revealed by of SMAD2 and SMAD3 (pSMAD2/3) phosphorylation in cells within areas of perivascular fibrosis (Figure 6 and Supplemental Figure 8). Cells positive for pSMAD3 were also positive for α–smooth muscle actin (α-SMA) and/or collagen or periostin (Figure 6A and Supplemental Figure 8), indicating that they were undergoing mesenchymal transition to myofibroblasts. Myofibroblasts produce excessive collagen in tissues undergoing pathological fibrosis (Figure 7) (28). HIV*-*Tg26 mice treated with atorvastatin had significantly lower levels of nuclear pSMAD2/3 translocation in cells coexpressing myofibroblast markers (Figure 6 and Supplemental Figure 8), suggesting that atorvastatin inhibits TGF-β1–induced signaling linked to fibrosis-related gene responses in vivo.

To identify additional cells contributing to mesenchymal transition, producing excessive collagen via induction of TGF-β1 signaling and fibrosis, we costained heart sections with markers specific to fibroblasts (PDGFβR), endothelial cells (CD31), and macrophages (CD206). HIV-Tg26 mice treated with RTV or TDF-FTC-DTG had more cells coexpressing these markers along with α-SMA, collagen, periostin, and pSMAD3. In comparison, atorvastatin-treated HIV*-*Tg26 mice showed fewer cells with such colocalized signals and had significantly lower nuclear pSMAD2/3 translocation (Figure 6, A–C, and Supplemental Figure 8). This suggests that atorvastatin inhibits TGF-β1–induced fibrotic gene responses in vivo. Correspondingly, collagen-positive areas reflecting myofibroblast transition were lower in atorvastatin-treated HIV-Tg26 hearts compared with their vehicle-treated counterparts (Figure 6). Additionally, TGF-β1–induced expression of myofibroblast marker genes α-SMA (*ACTA2*) and collagen type 1 alpha 1 (*COL1A1*) were blocked in atorvastatin-treated HIV-Tg26 mice (Supplemental Figure 5D).

Taken together, these data indicate that platelet-derived TGF-β1–mediated signaling in cardiac cells, including endothelial cells, fibroblasts, and macrophages, can trigger their transformation into excessive collagen-producing myofibroblasts, inducing cardiac fibrosis. These processes can be mitigated by atorvastatin or by inhibiting or depleting platelet TGF-β1 (Figure 7).

## Discussion

Our work investigating HIV/ART-induced cardiac pathology in mouse models of HIV infection yielded several findings of clinical relevance to accelerated HFpEF seen in PWH, providing insights into potential mechanisms. We previously showed that platelet-derived TGF-β1 is an important mediator of RTV-associated cardiac fibrosis in WT mice (22). Our study shows that a lower dose of RTV, as used in protease inhibitor–boosted ART regimens, but not the protease inhibitor DRV alone, and the INSTI-based regimen TDF-FTC-DTG, augment cardiac fibrosis and diastolic dysfunction in the context of HIV, above levels seen with HIV alone. The level of cardiac fibrosis in HIV/ART mice, 3%–4%, was equivalent to that estimated by imaging studies in ART-treated PWH (2%–3%) (29) and sufficient to induce diastolic dysfunction. It was accompanied by cardiac steatosis, also a feature of HFpEF in ART-treated PWH. Areas of ART-induced fibrosis in HIV/ART mice were accompanied by myofibroblast infiltration with elevated levels of SMAD2/3 phosphorylation, reflecting TGF-β1 signaling and excess collagen production. A role for platelet TGF-β1 in cardiac fibrosis associated with certain ART drugs was further supported by the correlation of plasma TGF-β1 with both fibrosis and diastolic dysfunction.

In terms of practical interventions, atorvastatin protected HIV-Tg26 mice, both untreated and exposed to either RTV or the INSTI-based cocktail TDF-FTC-DTG, from cardiac fibrosis and steatosis and diastolic dysfunction. These effects appear to be channeled, at least in part, through the inhibition of TGF-β1 signaling. They correlated with the presence of fewer myofibroblasts in the hearts of HIV/ART mice treated with atorvastatin versus vehicle. Our results are consistent with the known antioxidant/antiinflammatory effects of statins (30) and with atorvastatin’s ability to attenuate the rise in soluble ST2, a marker of fibrosis in PWH on ART (31). Although pitavastatin has a relatively stronger effect on HDL cholesterol than atorvastatin (32), the latter has other salutary effects. It can significantly reduce noncalcified coronary plaque volume relative to a placebo in PWH on ART, despite no change in HDL cholesterol (33). It also has an indirect immunomodulatory effect in the setting of HIV, increasing Tregs and inhibiting expression of the critical HIV coreceptor CCR5 (34). Finally, although potential drug-drug interactions with statins and ART are primarily based on theoretical considerations, caution is recommended using atorvastatin, but not pitavastatin, in the setting of protease inhibitor–based ART due to its effects on cytochrome CYP3A4 (35). However, nucleoside reverse transcriptase inhibitors (NRTIs) and INSTIs share an affinity for the breast cancer resistance protein drug transporter with pitavastatin, whereas no such interaction has been observed with atorvastatin (35). The choice of a specific statin for CVD prophylaxis in PWH on ART may need to be individualized based on these and other more traditional CVD risk considerations.

Our observations dovetail with recent work by Marunouchi et al. demonstrating that statins suppressed cardiac fibrosis and diastolic dysfunction in WT mice fed a high-fat diet and exposed to the nitric oxide synthetase inhibitor N[w]-nitro-l-arginine methyl ester hydrochloride as a chemical means of inducing HFpEF (36). Assessment of cardiomyocytes from statin-pretreated mice versus control mice also revealed decreased phosphorylation of SMAD and MAPK, enzymes downstream of TGF-β, reflecting reduced TGF-β signaling in those cells (36). Our in vitro data showing that atorvastatin dose-dependently inhibits TGF-β1–induced SMAD2/3 signaling and TGF-β–responsive profibrotic responses, including PAI-1 and α-SMA, are also consistent with studies in human cardiac fibroblasts demonstrating that atorvastatin suppresses SMAD and MAPK signaling (37). Our data further extend previous studies (27) indicating that atorvastatin can block platelet activation linked to standard agonists (ADP, collagen, and arachidonate) as well as certain cytokines. In our study, PPAR-γ, known to be affected by atorvastatin, albeit via an unknown mechanism, remained unchanged across groups and treatments (Supplemental Figure 9). It is thus plausible that statins act via a dual mechanism in HIV/ART-associated CVD characterized by HFpEF, blocking TGF-β1 release from platelets and subsequently inhibiting TGF-β1 signaling in cardiac cells.

In terms of the impact of atorvastatin on cholesterol and inflammation, 2 traditional risk factors for CVD, statins can lower inflammatory cytokines and LDL cholesterol in humans, but these effects did not correlate with the ability of a related lipophilic statin, pitavastatin, to affect atherosclerotic CVD in PWH on ART (2). Those results are paralleled by our mouse models, which showed no significant reduction in IL-6, TNF-α, or total cholesterol. We acknowledge that our data are not conclusive regarding whether atorvastatin’s effects are completely independent of the modulation of cholesterol metabolism.

Our focus on TGF-β and fibrosis should also be viewed in the general context of HIV/ART-linked CVD risk, both atherosclerotic and fibrosis-associated. In REPRIEVE, pitavastatin increased the abundance of PCOLCE, which enhances proteinases involved in vascular extracellular matrix production, favoring the transformation of plaques that are vulnerable to fragmentation to more stable coronary lesions (4). This is consistent with the salutary effects of TGF-β1 in the early stages of atherosclerosis (5). In later stages, when pathological fibrosis and HFpEF develop, suppression of TGF-β1 signaling may be beneficial. Both early and late stages of CVD in PWH might therefore benefit from statin intervention, albeit for distinct reasons. Although there are no validated tools to predict who among PWH receiving ART may clinically progress to HFpEF, the newly developed American Heart Association PREVENT HF risk score has proven valuable in a limited clinical study of such individuals (38).

The concomitant development of cardiac steatosis and fibrosis in our ART-treated HIV mice is also of interest. Among PWH in REPRIEVE, increased peri-coronary adipose tissue density was independently associated with the prevalence and severity of coronary plaque (39). However, the interplay between pathways leading to cardiac fibrosis and steatosis is incompletely understood. Although ART-treated PWH without heart failure have higher levels of cardiac fibrosis and steatosis compared with individuals without HIV, cardiac steatosis stands out as the structural pathology correlating most closely with diastolic dysfunction (10, 29, 40, 41). Cardiac magnetic resonance spectroscopy studies reveal excess intramyocardial lipid deposition among ART-treated PWH in association with reduced diastolic function (10, 40). Certain ART drugs are also associated with ectopic visceral and hepatic fat deposition (42). Our findings underscore the need for further exploration of HIV/ART-induced cardiac fat accumulation. Cardiac steatosis is an emerging global problem among ART-treated PWH (1, 19), paralleling rising rates of obesity in this population (43). Future studies could assess whether anti-obesity therapeutics known to confer cardioprotection, such as glucagon-like peptide 1 receptor agonists (GLP-1 RA) (44), reduce cardiac and other ectopic fat accumulation in PWH. In this regard, a pilot study of ART-treated PWH receiving the GLP-1 RA semaglutide revealed weight loss accompanied by a significant reduction in liver fat (45).

Our study has limitations. Based on our prior work with RTV, we focused our mechanistic experiments on TGF-β pathways leading to fibrosis. However, other pathways may also be relevant. In terms of the impact of HIV and ART on diastolic versus systolic function and links to fibrosis, we did find a small decrease in EF in HIV-Tg26 versus WT mice. But no further decrease occurred after ART treatment, and those EF values were still within the normal range for systolic heart function. However, our studies were terminated at 8 weeks; ART influence on EF might require longer periods, a subject for future investigation. Our work was strengthened by studying ART-induced cardiac pathology in 2 different mouse models of HIV and by interrogating ART’s effects in mice with platelet-specific TGF-β1 deletion to gain mechanistic insights. We also explored the impact of concomitant statin therapy, paving the way for clinical correlations. Selection among classes of ART drugs and individual drugs within classes, based on data presented here, could be considered. Apart from statins, suppression of ART-induced TGF-β1 release from platelets and its activation could be explored. This might be accomplished via antiplatelet agents, compounds that inhibit TGF-β1 release from platelets, and pharmacological inhibition of TGF-β signaling, such as with galunisertib (46).

## Methods

### Sex as a biological variable.

Both male and female mice were included in this study to account for sex as a biological variable.

### Mouse experiments.

Each mouse was numbered, and all experiments were performed by investigators blinded to mouse genotype. All mice used (aged 6–10 weeks) were housed in a controlled environment (23°C ± 2°C; 12-hour light/12-hour dark cycles) and fed a standard diet (PicoLab Rodent Diet). HIV-Tg26 is a transgenic mouse expressing 7 of the 9 HIV proteins under the control of a viral LTR promoter (16). They were bred with either FVB/NJ mice or C57BL/6 background for at least 10 generations. To create a platelet-specific KO of the *Tgfb1* gene on a Tg26 background, we crossed PF4CreTgfb1^fl/fl^ (TGF-β1^Platelet-Δ^) mice (28) with HIV-Tg26 mice to generate TGF-β1^Platelet-Δ^Tg26 mice. We confirmed the inactivation of the *Tgfb1*-floxed allele in platelets by genotyping and performed phenotypic characterization by measuring TGF-β1 levels in platelets, serum, and plasma of mice homozygous for TGF-β1^Platelet-Δ^Tg26. Tg26 littermates without Gp1bCre or Tgfb1^fl/fl^ were used as controls.

All mice infected with replication-competent HIV were housed in the animal facility of the Belfer Research Building at Weill Cornell Medical College. Humanized HIV-PDX mice were generated by engrafting memory CD4^+^ T cells into NSG mice (NOD/SCID IL2rγ^−/−^, The Jackson Laboratory, 00557). Memory CD4^+^ T cells were isolated from PBMCs of a PWH on ART (17). On day 35 after engraftment, mice received memory CD8^+^T cells from the same donor and were then i.v. inoculated with 10,000 tissue culture ID_50_ of the HIV-1 JRCSF isolate. From day 84, mice were treated s.c. with 100 μL of vehicle or a combination ART regimen consisting of TDF (57 mg/kg), FTC (143 mg/kg), and DTG (7 mg/kg) (47), or single protease inhibitors RTV (5.5 mg/kg) and DRV (16.2 mg/kg) for 8 weeks.

### ART drug dosing.

Dosages of ART drugs paralleled those in humans, as documented by both pharmacokinetic/pharmacodynamic (PK/PD) data in mice (48) and measurements of ART drug concentrations in lymph nodes of humanized mice versus humans, showing equivalent tissue levels (49). Notably, our ART involved much lower concentrations than those used in many published rodent models of HIV infection (50), where some effects may have been influenced by supratherapeutic dosing.

### Collection and preparation of mouse serum, plasma, and platelet releasate.

To obtain serum, whole blood was collected by retrobulbar puncture into tubes without an anticoagulant and incubated at 37°C for 4 hours, then centrifuged (13,000*g*, 20 minutes, 4°C) to collect the supernatant. To obtain plasma, blood was collected by retrobulbar puncture into a polypropylene tube containing 0.1 volume of 3.8% sodium citrate and PGE_1_ (1 μM, pH 7.4) and immediately centrifuged at 12,000*g* for 5 minutes at 4°C. All samples were stored at –80°C until analysis. Washed platelets were prepared as previously described (51). Platelets (0.5 × 10^9^) were stimulated with the drug for 10 minutes at 37°C, and releasates were collected as the supernatants after sample centrifugation at 13,000*g* for 15 minutes at 4°C.

### Measurement of TGF-β1 levels and signaling.

Total TGF-β1 in platelets, serum, and plasma was measured using a DUO-antibody ELISA specific for the activated form of TGF-β1 (R&D Systems) after converting latent TGF-β1 to its active form by acidification followed by neutralization. TGF-β1–induced signaling was measured using a functional bioassay with the MLEC cell line stably expressing a luciferase reporter gene under the control of the PAI1 promoter (24). Briefly, MLECs (2.5 × 10^4^) were plated in a 96-well tissue culture plate and allowed to adhere for 3 hours. The medium was replaced with 90 μL of serum-free DMEM containing antibiotics, and 10 μL of the test sample was added and incubated for 16–18 hours at 37°C. PAI1-driven luciferase activity was assayed from cell lysates in an automated luminometer using a luciferase assay system (Promega). The MLEC assay was used to confirm that active TGF-β1 was driving the signaling response. We also assessed TGF-β1 signaling via SMAD activation by stimulating mouse endothelial cells or MLECs with active TGF-β1 and immunoblotting for SMAD2/3 phosphorylation using a mAb specific for phosphorylated Smad2/3. In some experiments, samples were incubated with a TGF-β1 neutralizing antibody or atorvastatin to assess the specificity of TGF-β1 signaling detected by the PAI1 luciferase and Smad2 phosphorylation assays.

### Assessment of cardiac function.

Systolic and diastolic function were measured using a high-resolution ultrasound system (Vevo 2100, VisualSonics) following established methods (51). Systolic function parameters were assessed at end-diastole and end-systole using M-mode images acquired at the level of the papillary muscles in a left parasternal short-axis view. Left ventricular EF and fractional shortening were calculated. Diastolic function indices were recorded in an apical 4-chamber view sound window using pulse-wave or tissue Doppler echocardiography to measure E and A wave peak velocities. A′- and E′- tissue Doppler waves and E/A ratios were calculated following the method previously described (51).

### Histology.

Animals were euthanized, and their hearts were excised, perfused with saline, weighed, and fixed in 4% paraformaldehyde. Myocardial fibrosis was evaluated by staining with H&E, Masson’s trichrome, and Picrosirius red. The degree of fibrosis was graded on a scale of 0 to 4 by an expert veterinary pathologist unaware of the treatment, using methods established in our lab (51). Masson’s trichrome–stained images were acquired at high magnification using an Aperio Slide Scanner, and Picrosirius red–stained images were acquired using a polarized scanner. An artificial intelligence deep learning method was adapted to develop an algorithm for quantification (52) (Supplemental Figure 1).

### Oil Red O staining for cardiac fat/lipid.

Heart sections were fixed in 4% paraformaldehyde for 24 hours, followed by optimal cutting temperature (OCT) mounting and cryo-sectioning at 6 μm thickness. They were stained with Oil Red O (MilliporeSigma), following the manufacturer’s protocol. Whole heart sections were scanned using an Aperio Slide Scanner and assembled for quantification using ImageScope software.

### Immunostaining and confocal imaging.

Immunofluorescence-stained paraffin-embedded heart sections (5–6 μm) were brought to room temperature or deparaffinized and washed in PBS. Antigens were retrieved in citrate buffer (pH 6.0, Sigma-Aldrich) using a water bath (95°C for 10–12 min) or an antigen retrieval system (Electron Microscopy Science). Heart sections were washed with 0.1% Triton X-100 in PBS (PBST), incubated for 1 hour in blocking buffer (1% BSA in 0.1% PBST), and then stained with primary antibody (Supplemental Table 1) for 2 hours at room temperature or overnight at 4°C. Heart sections were then washed 3 times in PBST, incubated with the secondary antibody (Supplemental Table 1) in blocking buffer for 1 hour, washed again with PBST, and mounted with Alexa Fluor G reagent with DAPI. Images were captured with a confocal microscope using 20× or 40× objectives and visualized as 3D projections in Imaris software. For IHC, heart tissue sections were deparaffinized/rehydrated, antigens were retrieved using citrate buffer, and nonspecific binding was blocked with blocking buffer. Sections were incubated with primary antibodies against perilipin, phosphorylated SMAD2 and SMAD3, PDGFβR, α-SMA, CD31, CD206, PPAR-γ, and collagen (Supplemental Table 1).

### Real-time PCR.

Total RNA was extracted from cells using the RNeasy Mini kit (QIAGEN). cDNA was prepared from the RNA using the High-Capacity RNA-to-cDNA kit (Applied Biosystems), and real-time PCR was performed with ready-made primer sets for mouse *ACTA2* and *COL1A1* on a Bio-Rad real-time PCR system. Thermal cycling conditions were as follows: 50°C for 2 minutes, followed by 95°C for 10 minutes, 95°C for 15 seconds, and 60°C for 1 minute. A total of 40 cycles were run. Data were normalized to the endogenous control gene *GAPDH*.

### Statistics.

Statistical analysis was performed using GraphPad Prism, SAS version 9.4. Multiple linear regression models were used for fibrosis and diastolic dysfunction. Comparisons were made using the Kruskal-Wallis test, pairwise 2-sample Wilcoxon’s rank-sum tests, and 2-tailed *t* tests. All data are expressed as mean ± SD or SEM. Differences were considered statistically significant at *P* less than 0.05.

### Study approval.

Mouse experiments were conducted in accordance with the *Guide for the Care and Use of Laboratory Animals* (National Academies Press, 2011) and were approved in advance by the IACUCs of Oklahoma Medical Research Foundation, Oklahoma City, Oklahoma, USA, and Weill Cornell Medical College, New York, New York, USA.

## Author contributions

KS, DB, BC, TAA, TV, TW, SG, AD, and IGM designed the mouse models of HIV/ART-induced pathology and performed the experiments and collected and analyzed the data. SC analyzed the data and performed statistical modeling. PK, BRJ, and KMF provided essential conceptualization reagents/samples. JL and JA conceived the idea and drafted the manuscript. JA supervised the project, and all authors contributed to the critical revision and final approval of the text. JA, JL, KS, DB, TW, BRJ, PK, and BC designed the research protocol. KS, DB, TW, BC, TAA, TV, SG, AD, and IGM performed the research. KS, DB, TW, KMF, and SC analyzed the data. JA and JL acquired funding and wrote the paper with clinical conceptualization.

## Conflict of interest

The authors have declared that no conflict of interest exists.

## Funding support

- HL167656 National Institutes of Health (NIH) / National Heart, Lung, and Blood Institute (NHLBI (to JA).
- HL148123 NIH / NHLBI (to JA).
- Angelo Donghia Foundation (to JL).

## Supplementary Material

Supplemental data

Supporting data values

## Figures and Tables

**Table 1:**
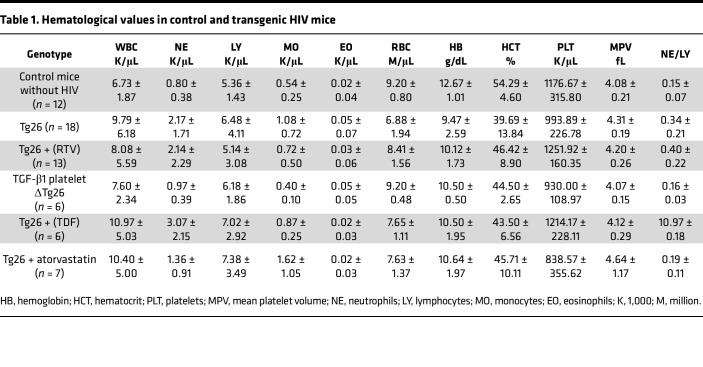
Hematological values in control and transgenic HIV mice

## Version 1 02/06/2026

In-Press Preview

## Version 2 07/08/2026

Electronic publication
