# Supplementary material for: Atorvastatin suppresses HIV/antiretroviral drug–induced cardiac fibrosis and dysfunction in mice by blocking platelet TGF-β1 signaling
Source: JCI Insight. 2026 Feb 5;11(13):e198647. doi: 10.1172/jci.insight.198647 (PMC13460851; doi:10.1172/jci.insight.198647)
Supplement: Supplementary file 1 [file jciinsight-11-198647-s030.pdf]

Supporting Information

Atorvastatin suppresses HIV/antiretroviral drug-induced cardiac fibrosis and dysfunction in mice by blocking platelet TGFβ1 signaling

Kumar Subramani[a], Denys Babii[a], Brienne Cole[a], Tayyab A. Afzal[a], Thamizhiniyan Venkatesan[a], Trevor Word[a], Sandra Gostynska[a], Sixia Chen[a,b], Kar-Ming Fung[a,b], Ali Danesh[c], Itzayana G. Miller[c], Paul Klotman[d], Brad R. Jones[c], Jeffrey Laurence[e], and Jasimuddin Ahamed[a,1]

[a]Cardiovascular Biology Research Program, Oklahoma Medical Research Foundation, Oklahoma City, OK 73104; [b]University of Oklahoma Health Sciences Center, Oklahoma City, OK 73104; [c]Division of Infectious Diseases, Weill Cornell Medical College, New York, NY 10065; [d]Baylor College of Medicine, Houston, TX 77030; and [e]Division of Hematology and Medical Oncology, Weill Cornell Medical College, New York, NY 10065.

Running Title: Role of platelet TGFβ1 in organ fibrosis

[1]To whom correspondence may be addressed. Jasimuddin Ahamed, Ph.D.
Email: ahamedj@omrf.org.

Fig. S1A

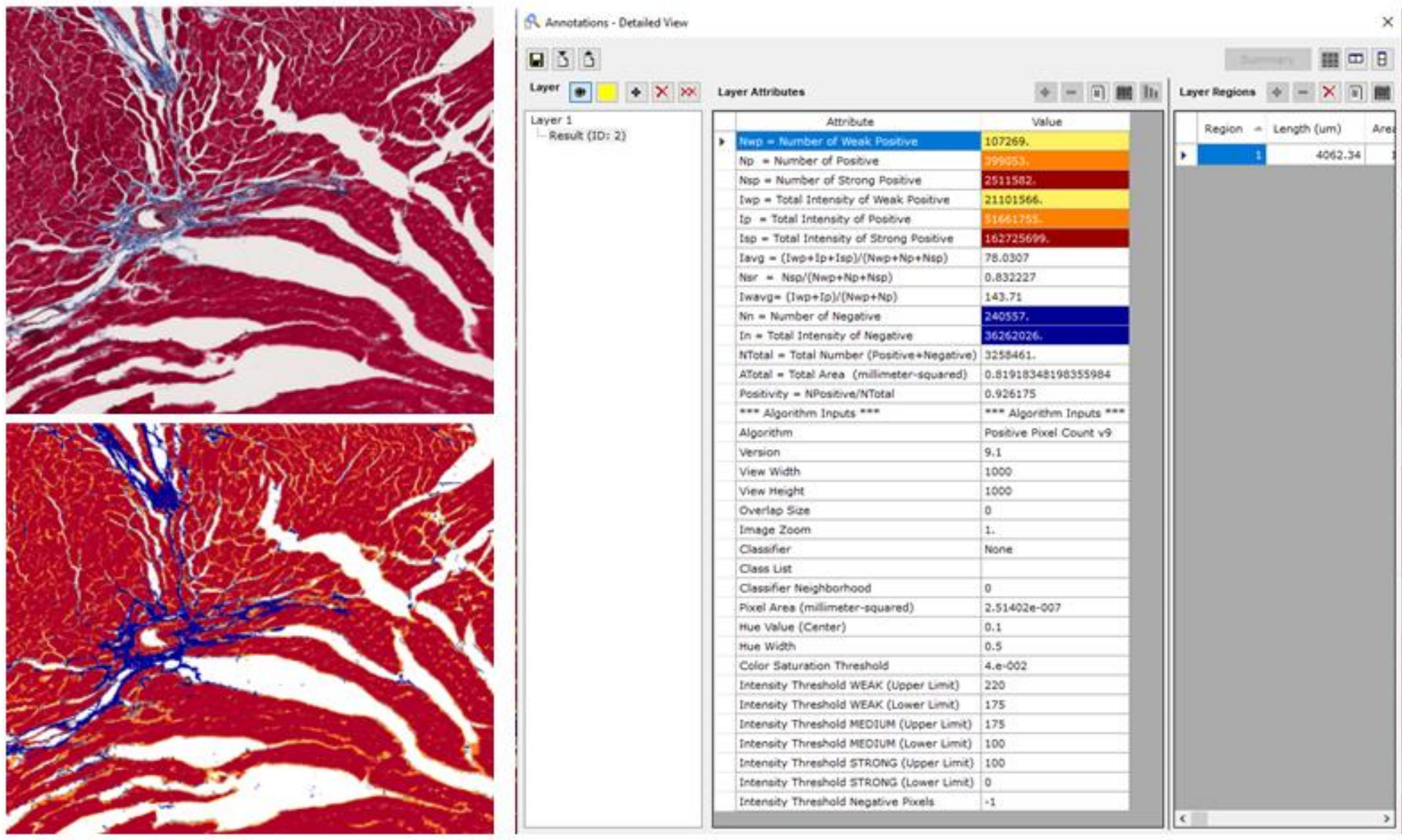
Annotations - Detailed View
Layer
Layer Attributes
Layer Regions
Summary
Layer 1
Result (ID: 2)
Attribute
Value
Nwp = Number of Weak Positive 107269.
Np = Number of Positive 399053.
Nsp = Number of Strong Positive 2511582.
Iwp = Total Intensity of Weak Positive 21101566.
Ip = Total Intensity of Positive 51661755.
Isp = Total Intensity of Strong Positive 162725699.
Iavg = (Iwp+Ip+Isp)/(Nwp+Np+Nsp) 78.0307
Nsr = Nsp/(Nwp+Np+Nsp) 0.832227
Iwavg= (Iwp+Ip)/(Nwp+Np) 143.71
Nn = Number of Negative 240557.
In = Total Intensity of Negative 36262026.
NTotal = Total Number (Positive+Negative) 3258461.
ATotal = Total Area (millimeter-squared) 0.81918348198355984
Positivity = NPositive/NTotal 0.926175
*** Algorithm Inputs *** *** Algorithm Inputs ***
Algorithm Positive Pixel Count v9
Version 9.1
View Width 1000
View Height 1000
Overlap Size 0
Image Zoom 1.
Classifier None
Class List
Classifier Neighborhood 0
Pixel Area (millimeter-squared) 2.51402e-007
Hue Value (Center) 0.1
Hue Width 0.5
Color Saturation Threshold 4.e-002
Intensity Threshold WEAK (Upper Limit) 220
Intensity Threshold WEAK (Lower Limit) 175
Intensity Threshold MEDIUM (Upper Limit) 175
Intensity Threshold MEDIUM (Lower Limit) 100
Intensity Threshold STRONG (Upper Limit) 100
Intensity Threshold STRONG (Lower Limit) 0
Intensity Threshold Negative Pixels -1
Region
Length (um)
1
4062.34

Fig. S1B

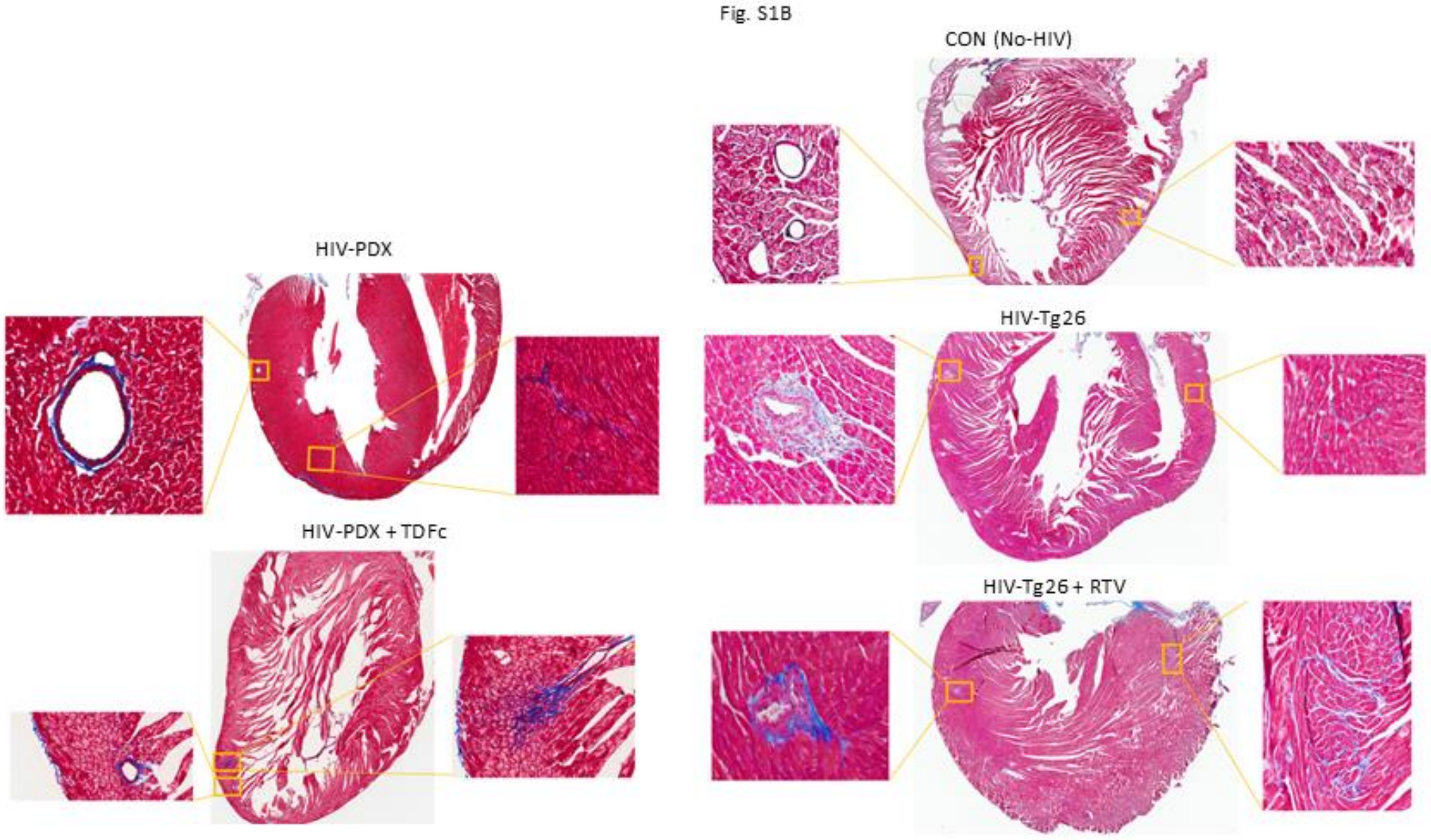

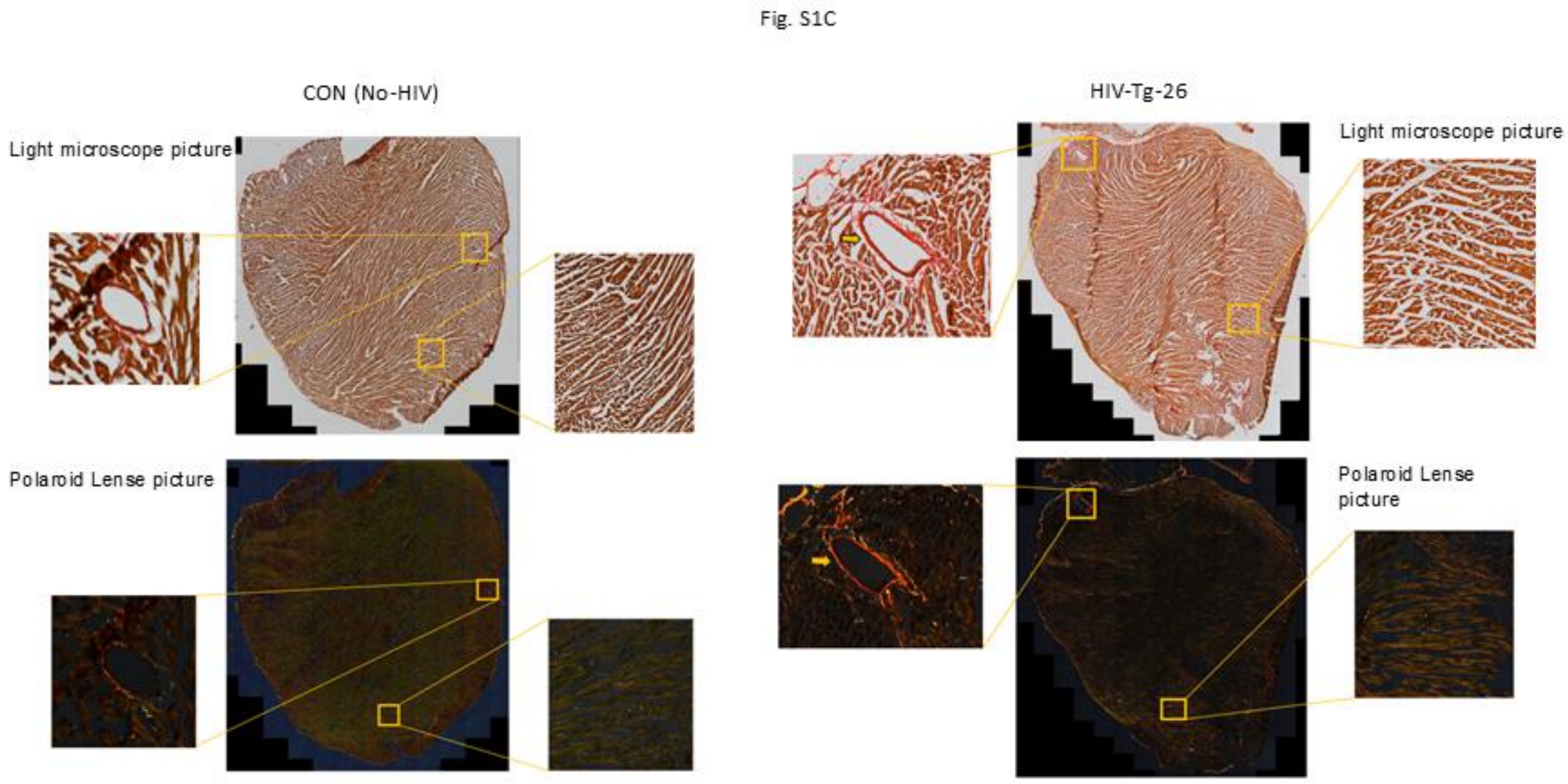

Fig. S1.

(**A**) Upper panel: Masson trichome-stained heart sections showing collagen (blue) in areas of fibrosis. For quantification of fibrosis, images of whole heart sections were used to identify focal fibrosis after removing heart valve-associated collagen staining. Lower panel: screenshot from ImageJ showing the algorithm-based calculation of the percentage of fibrotic regions within the total area of each heart section. Images of whole heart sections along with magnified areas of perivascular and interstitial fibrosis, stained with Masson trichome (**B**) and picrosirius red (**C**).

Fig. S2

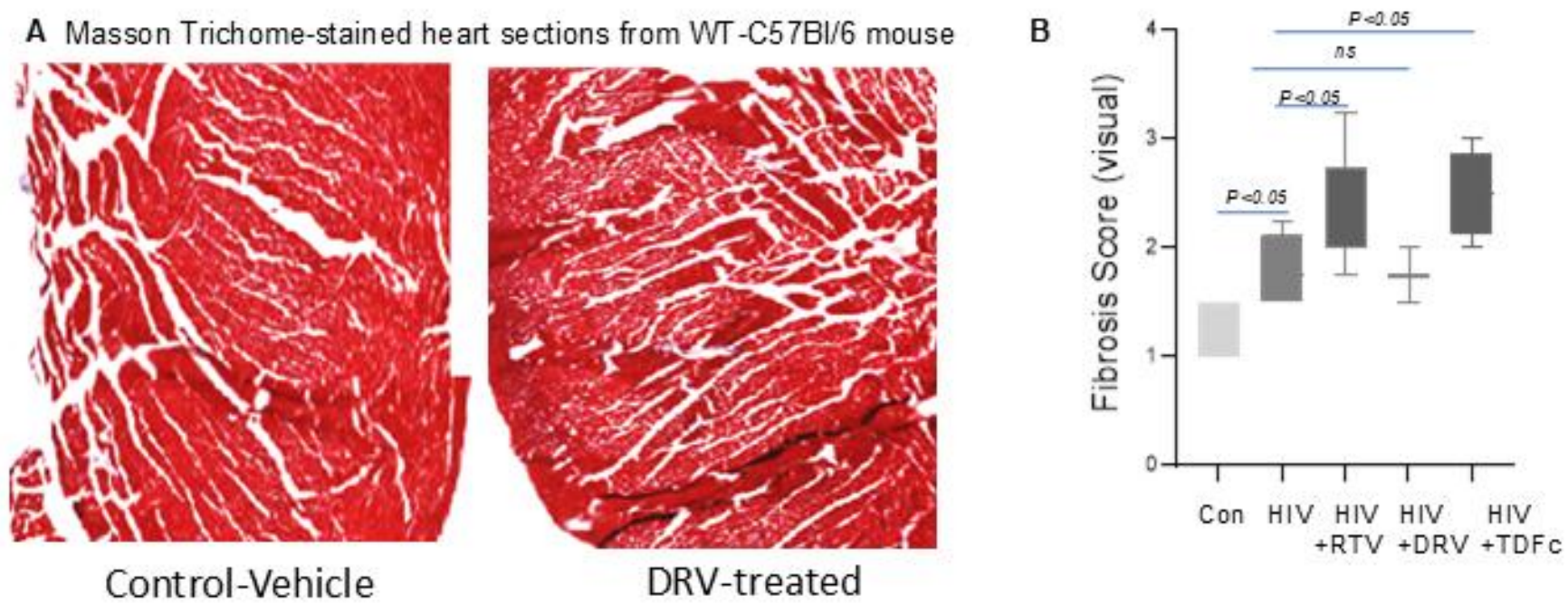

Fig. S2.

(**A**) Representative images of Masson trichome-stained heart sections from vehicle-treated control mice and darunavir (DRV)-treated C57Bl/6 mice. (**B**) Blind scoring by pathologists, showing elevated levels of cardiac fibrosis in HIV-Tg26 mice treated with ritonavir (RTV) or the tenofovir-emtricitabine-dolutegravir cocktail (TDF-FTC-DTG; TDFc), but not with DRV, compared with untreated controls.

Fig. S3

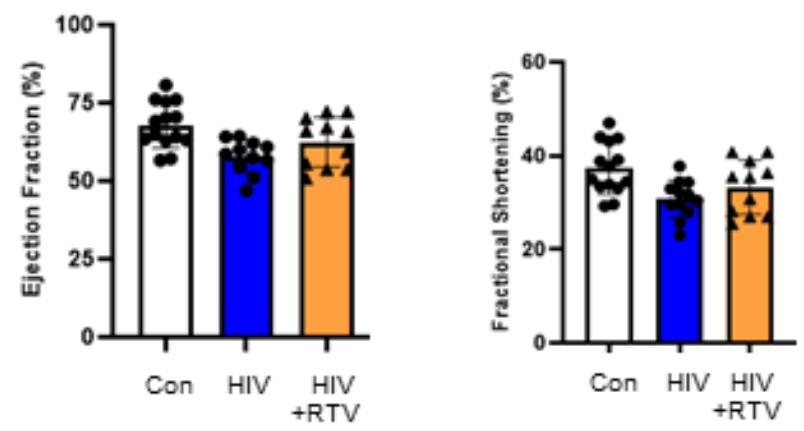

Fig. S3.
Parameters of systolic heart function, ejection fraction (EF) and fractional shortening (FS), in control mice and HIV-Tg26 mice treated with ritonavir (RTV) for eight weeks. EF and FS values were calculated from M-mode echocardiograms acquired using a Vevo 2100 ultrasound device and analyzed with FUJIFILM-VisualSonics cardio software.

Fig. S4

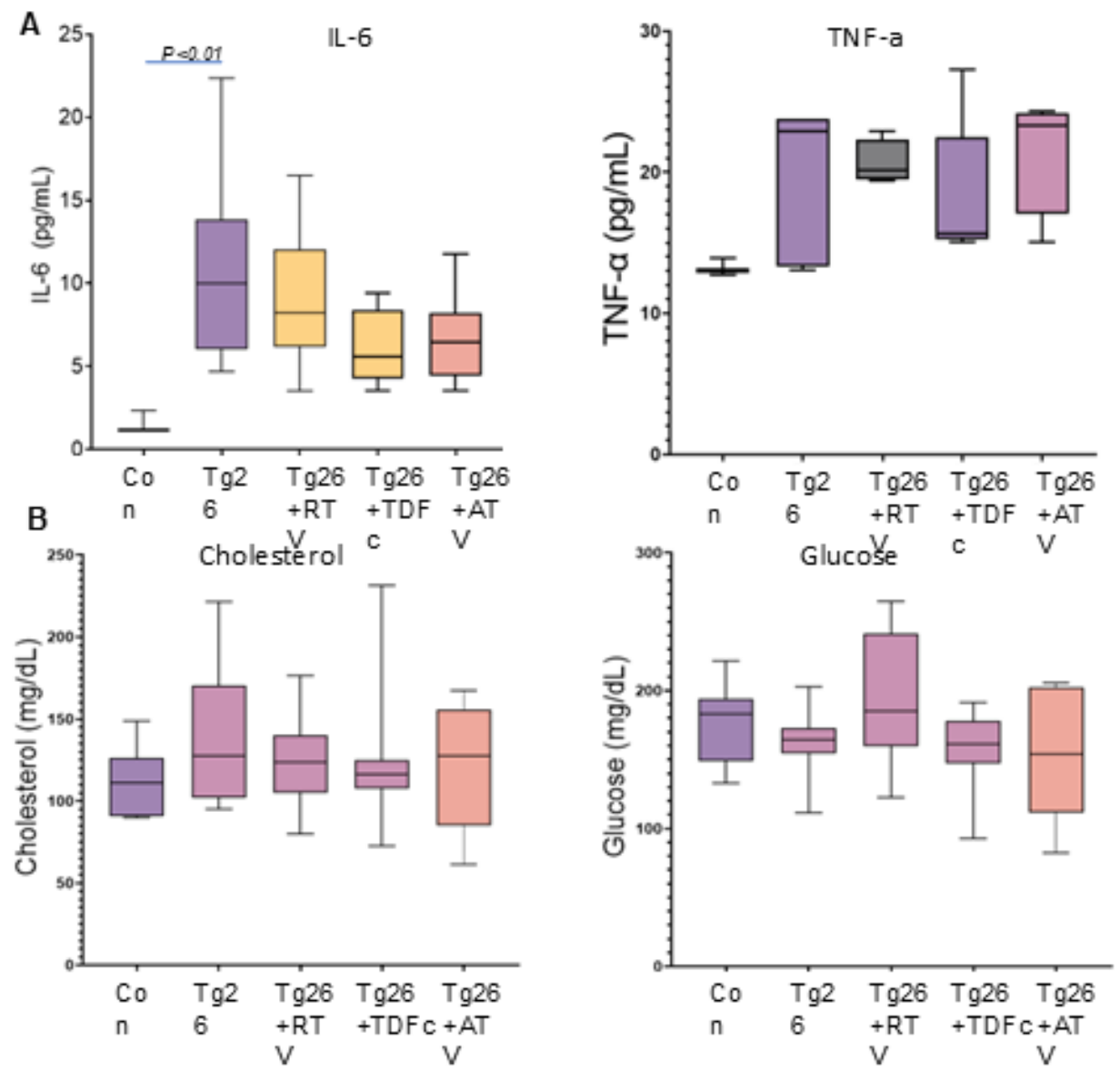

Fig. S4.

(**A**) Boxplots showing plasma IL-6 (left) and TNFα (right) levels, measured by ELISA, in control mice (No HIV) and HIV-Tg26 mice, without and with eight weeks of antiretroviral therapy (ritonavir (RTV) or the tenofovir-emtricitabine-dolutegravir cocktail (TDFc)), or atorvastatin (ATV) treatment. (**B**) Boxplots showing plasma total cholesterol (left) and glucose (right) levels, measured using colorimetric assays.

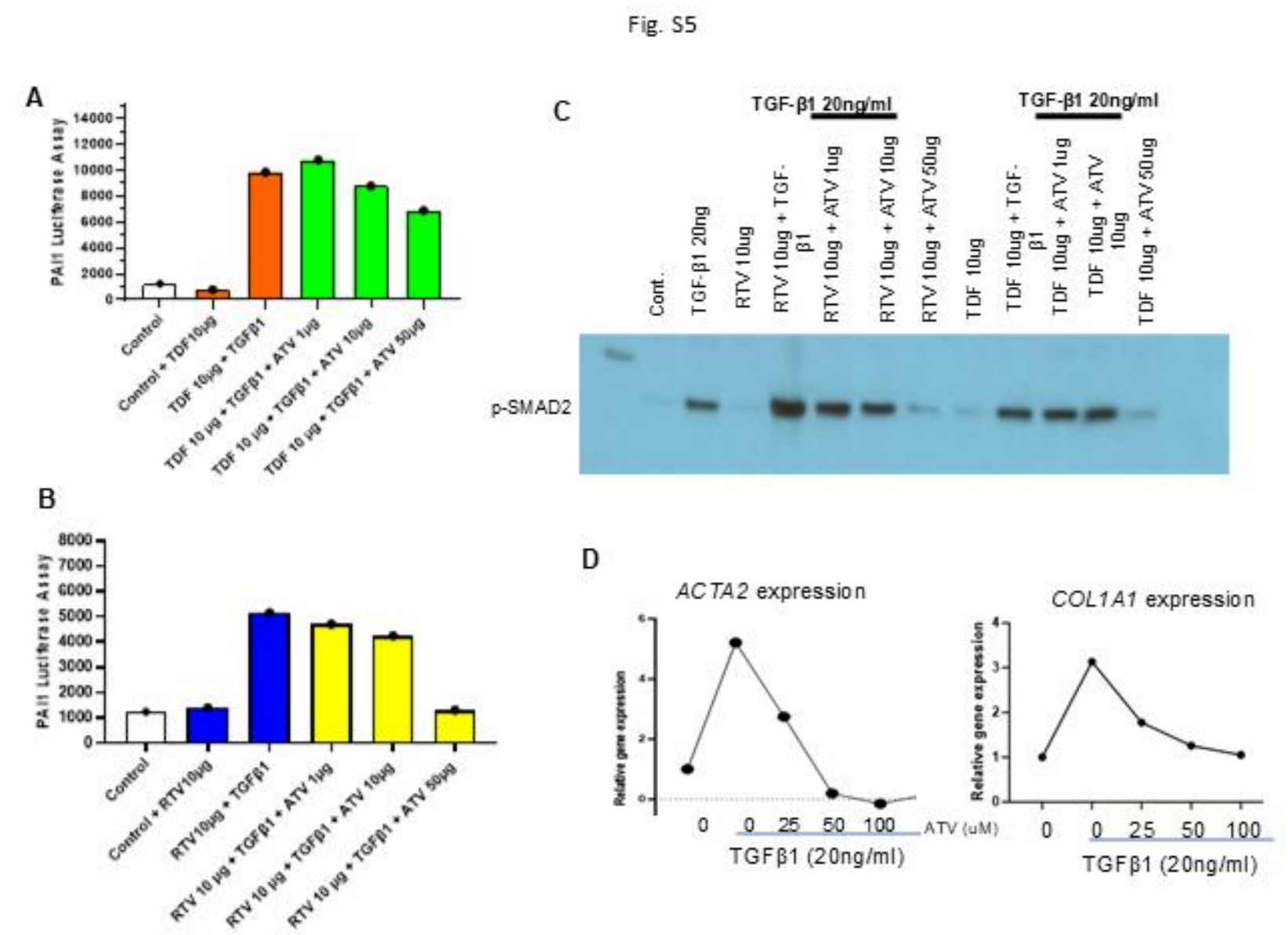

Fig. S5.

(**A, B**) Atorvastatin (ATV) inhibited TGFβ1 plus ritonavir (RTV)- or tenofovir-emtricitabine-dolutegravir cocktail (TDFc)-mediated signaling in a dose-dependent manner, as determined by PAI-1 luciferase activity, and (**C**) by pSMAD2 phosphorylation. (**D**) ATV also inhibited the expression of *ACTA2* and *COL1A1* genes, as determined by real-time polymerase chain reaction in endothelial cells stimulated with platelet-derived TGFβ1 for three hours.

Fig. S6

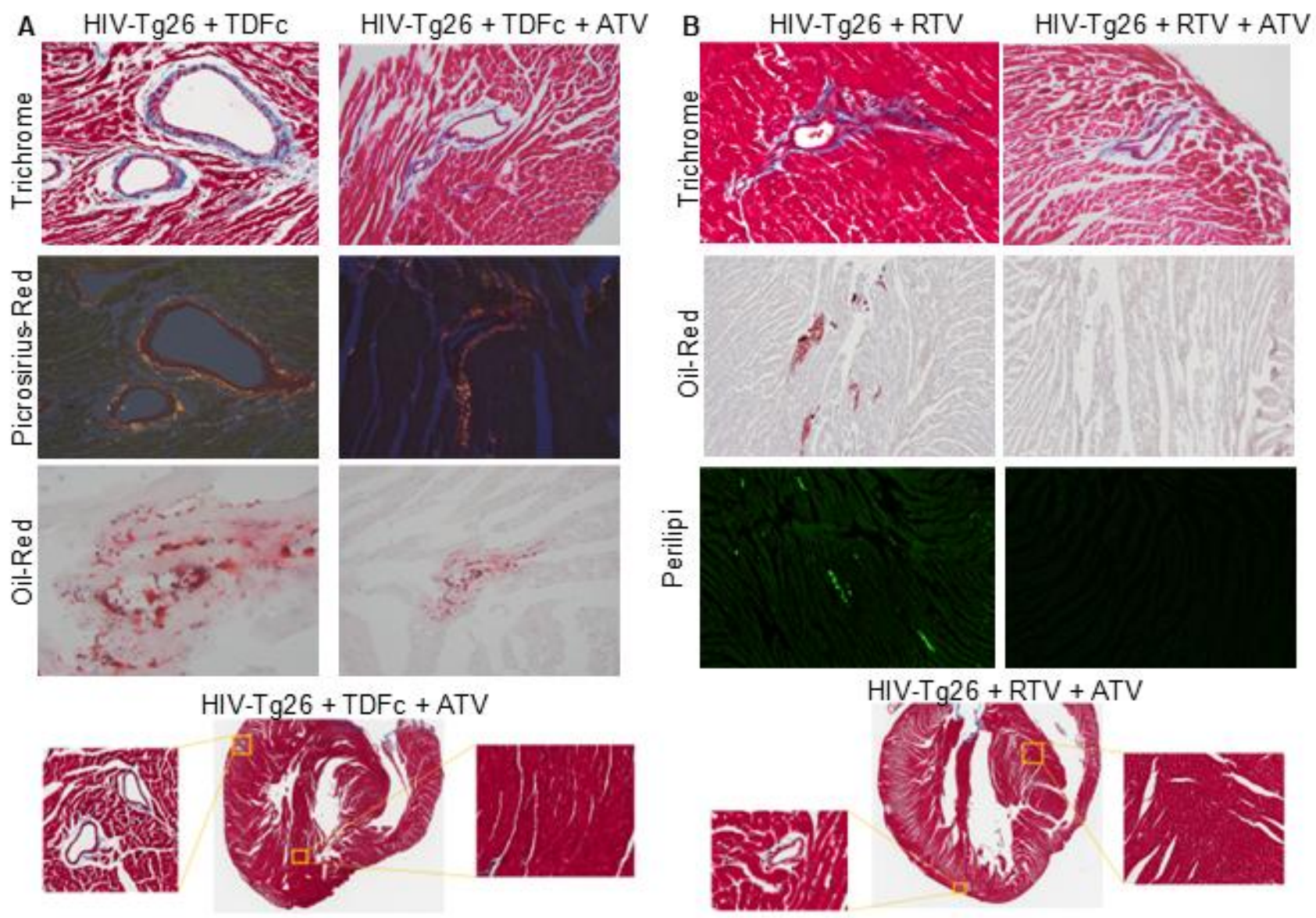

Fig. S6.

Atorvastatin (ATV) inhibited cardiac fibrosis and the deposition of lipid/fat cells, as observed by Masson trichome, picrosirius red, Oil Red, and Perilipin staining in HIV-Tg26 mice co-treated with (**A**) the tenofovir-emtricitabine-dolutegravir cocktail (TDFc) or (**B**) ritonavir (RTV). Lower panels show images of Masson trichrome-stained whole heart sections with perivascular and interstitial fibrosis.

Fig. S7

Effect of ATV on ART-induced platelet activation and TGFβ1 release

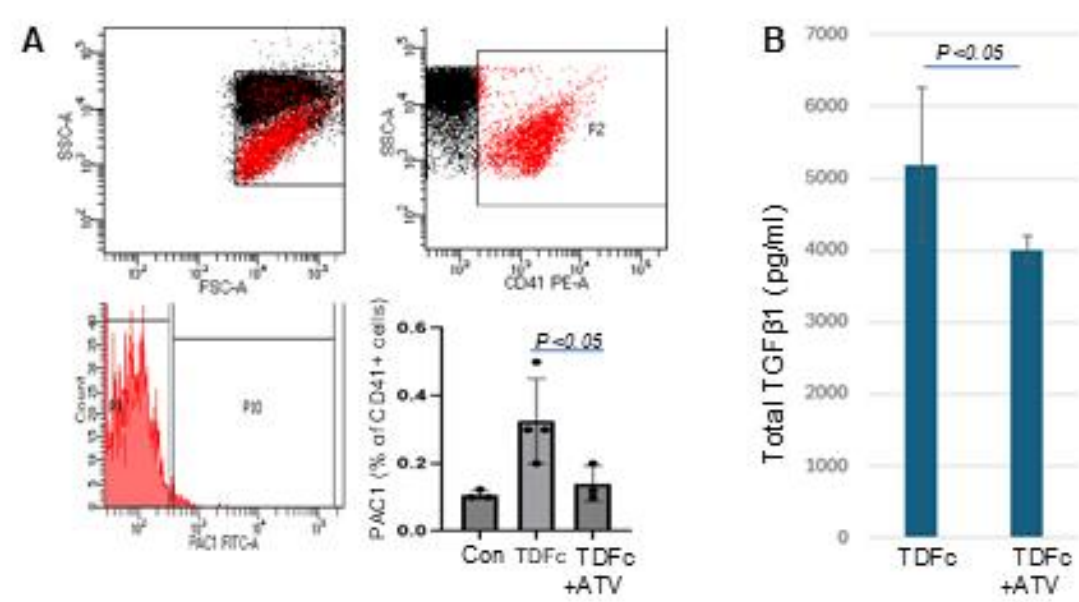

Fig. S7.
(**A**) Human platelet activation by the tenofovir-emtricitabine-dolutegravir cocktail (TDFc) before and after atorvastatin (ATV) treatment, measured by flow cytometry, gating on CD41-positive platelets expressing PAC1. (**B**) Total plasma TGFβ1 levels in HIV-Tg26 mice exposed to TDFc alone or co-treated with TDFc and ATV.

Fig. S8

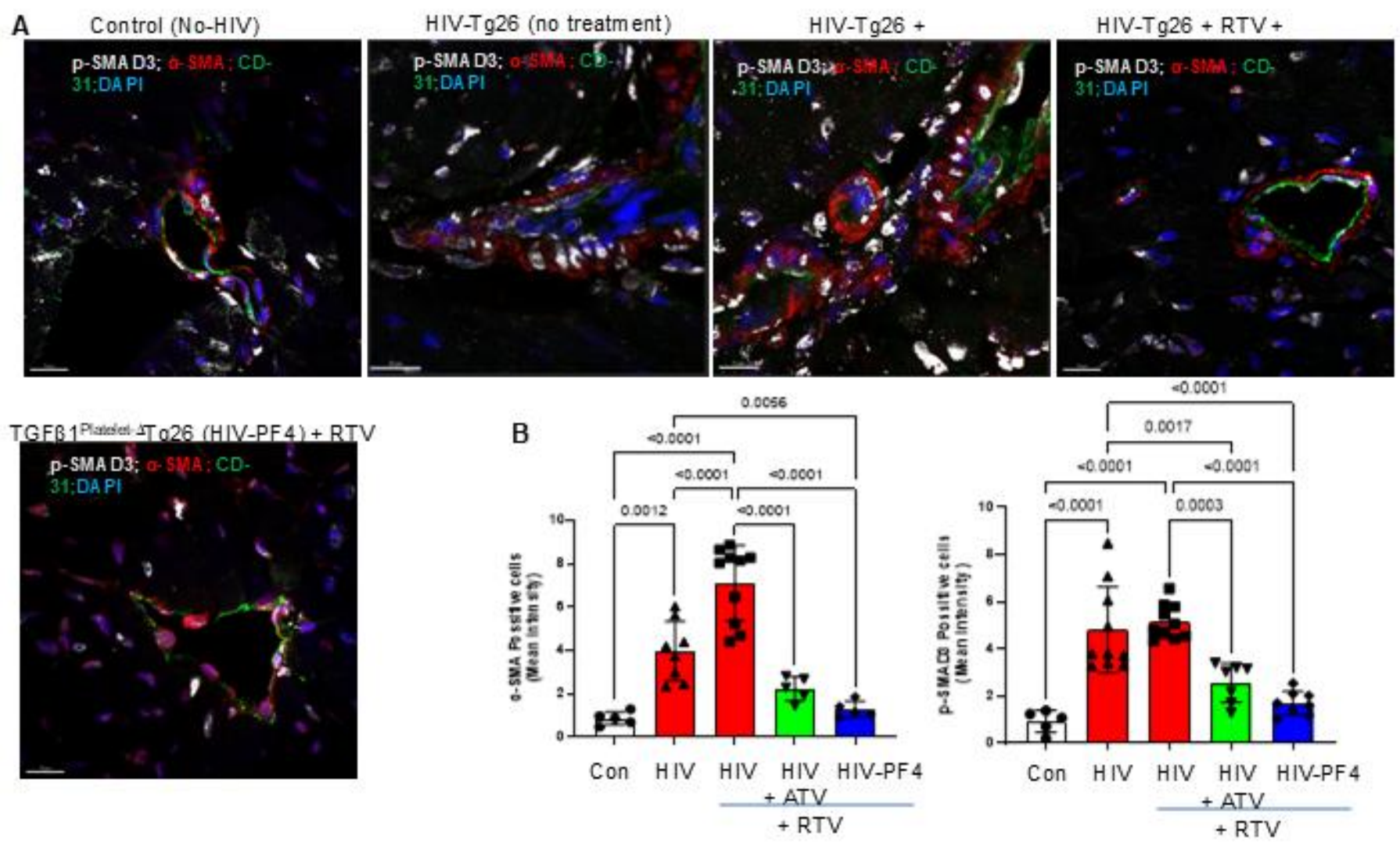

Fig. S8.

Atorvastatin (ATV) inhibits SMAD signaling. (**A**) Representative confocal images showing phosphorylation levels of SMAD3 (white) in endothelial cells (stained with CD31 antibody; green) and in mesenchymal and smooth muscle cells (stained with α-SMA antibody; red) in the hearts of control mice (no HIV), HIV-Tg26 mice treated with ritonavir (RTV) alone or co-treated with RTV and atrovastatsin (ATV), and PF4CreTgfb1-floxed (HIV-Tg26-PF4) mice. (**B**) Barplots showing the quantification of TGFβ signaling, as assessed by pSMAD3 staining intensity. The number of α-SMA- (left) or pSMAD3-positive (right) cells in PF4CreTgfb1-floxed (HIV-Tg26-PF4) mice is lower than in control mice, indicating lower mesenchymal transition and TGFβ signaling.

Fig. S9

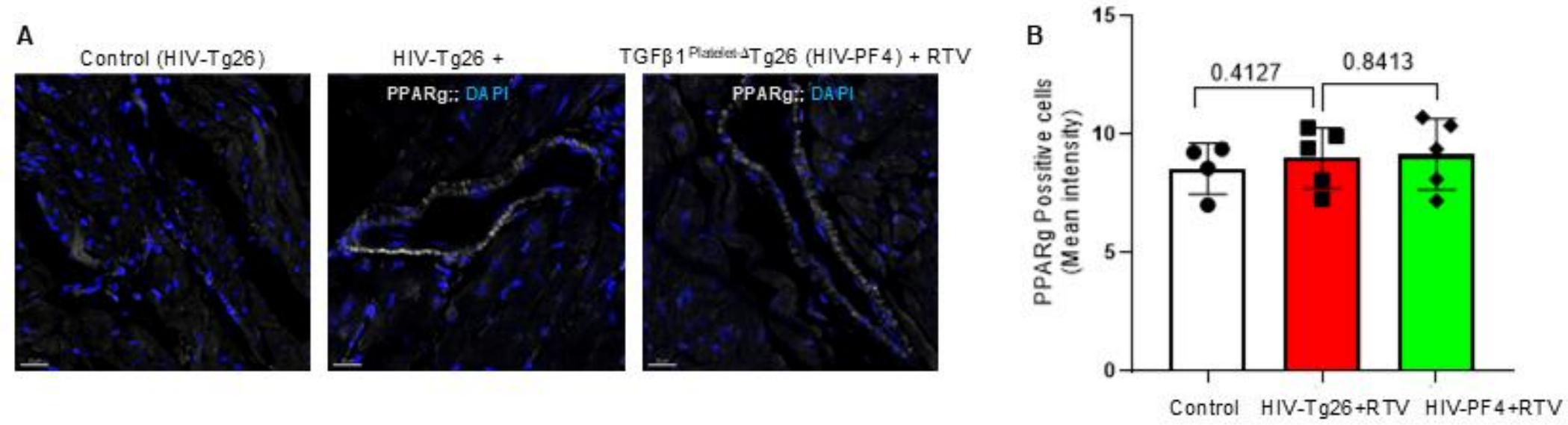

Fig. S9.

(**A**) Immunofluorescence signals of PPAR-gamma (white) visualized by staining with anti-PPAR-gamma antibody (white). (**B**) Barplot showing no difference in PPAR-gamma levels between groups and treatments. Groups of mice are:Control (HIV-Tg26) no treatment, HIV-Tg26 treated with RTV, HIV-Tg26-PF4CreTGFb1-flox mice treated with RTV.

List of antibodies and reagents

Antibodies/reagents	Vender	Cat No:	Dilution	Species	Note
Anti-pSMAD2	Millipore	AB3849	1:1000	Rabbit	Western blotting & Immunostaining
Anti SMAD2/3	Cell Signaling	8685	1:1000	Rabbit	Western blotting
Anti-pSMAD3	Rockland	600-401-919S	1:1000<br>1:250	Rabbit	Western blotting & Immunostaining
Colllagen-1	Sigma	SB1402151	1:1000	Mouse	Immunostaining
Alpha -SMA	Thermofisher	904601	1:250	Mouse	Immunostaining
CD206	Agilent	AF-2535	1:250	Goat	Immunostaining
Perilipin-1 (K117)	Cell Signaling	3467S	1:250	Anti-rabbit	Immunostaining
Donkey anti-Rabbit Alexa Fluor 647-conjugated	Jackson Immuno Research	711-607-003	1:250	Anti rabbit	Secondary antibody for IF
Donkey anti-goat Alexa Fluor 488-conjugated	Jackson Immuno Research	705-546-147	1:250	Anti goat	Secondary antibody for Immunostaining
Donkey anti-rat Alexa Fluor 594-conjugated	Jackson Immuno Research	712-586-150	1:250	Anti rat	Secondary antibody for Immunostaining
Donkey anti-mouse Alexa Fluor 594-conjugated	Jackson Immuno Research	715-586-150	1:250	Anti mouse	Secondary antibody for Immunostaining
PPAR-gamma (C26H12)	Cell Signaling	2435S	1:250	Rabbit	Immunostaining
Wheat Germ Agglutinin (WGA)	Thermofisher	W11262	1:1000	Alexa Fluor 594 Conjugate	Immunostaining
Goat anti-Rabbit, HRP-conjugated	Jackson Immuno Research	711-035-152	1:10000	Anti rabbit	Secondary antibody for Western blotting
Protein ladder	Bio-Rad	161-0374			
TGF beta1	Peprotech	100-21-2ug	20ng/mL		
Oil Red stain kit	Newcomer Supply INC	1277B			Paraffin section or Cryosection
Picrosirius Red Stain kit	IHC World	3012			Paraffin section or Cryosection
Masson Trichrome Stain kit	Electron Microscopy Science	26367-01	Bouin'S Fixative		Paraffin section or Cryosection
		26367-02	Weigerts Iron Hematoxylin A		
		26367-03	Weigerts Iron Hematoxylin B		
		26367-04	Biebrich Scarlet Solution 1%,		
		26367-05	Phosphomolybdic/Phosphotungstic Acid Soltn		
		26367-06	Aniline Blue Solution		
		26367-06	Acetic Acid 1% Aqueous		
SYBR™ Select Master Mix	Applied Biosystems™ Thermofisher	44-729-08	RT-PCR		
High-Capacity RNA-to-cDNA™ Kit	Applied Biosystems™ Thermofisher	43-874-06	RT-PCR		
Primers for RT-PCR	IDT	Col1A1 F	5-GAAACCCGAGGTATGCTTGA-3		
		Col1A1 R	5-GTTGGGACAGTCCAGTTCTT-3		
		ACTA2 F	5-CCTTCGTGACTACTGCCGAG-3		
		ACTA2 R	5-AATGCCTGGGTACATGGTGG-3		

		GAPDH F	5-GGCAAATTCAACGGCACAGT-3
		GAPDH R	5-CGCTCCTGGAAGATGGTGAT-3
